# Deterministic nowcasting of geostationary satellite infrared brightness temperature using 3D U-Net diffusion model

**DOI:** 10.1038/s41598-025-34207-9

**Published:** 2026-01-03

**Authors:** Vesta Afzali Gorooh, Luca Delle Monache, Duncan Axisa, Agniv Sengupta, Zhenhai Zhang, Fred Martin Ralph

**Affiliations:** Center for Western Weather and Water Extremes, Scripps Institution of Oceanography, University of California, San Diego, La Jolla, CA USA

**Keywords:** Climate sciences, Mathematics and computing

## Abstract

We present a generative modeling approach for nowcasting infrared (IR) brightness temperatures (Tb) from geostationary satellite observations (~ 10.8 μm) that couples a denoising diffusion probabilistic model with a 3D U-Net backbone. Using SEVIRI observations, the model ingests six hours of IR history and produces six-hour nowcasts at 15-min resolution. Deterministic evaluation is performed on an independent July–September 2022 test set and benchmarked against 3D U-Net, ConvLSTM, and Optical Flow extrapolation baselines. The diffusion model enhances prediction accuracy, yielding lower errors and higher correlation than all baselines across most forecast lead times, with a persistent advantage through the 2-hour lead time and reduced but still evident gains at longer leads. We complement the statistical assessments with a perceptual and a probabilistic diagnostic; diffusion achieves the highest SSIM at all leads and the lowest CRPS among all models (CRPS generates MAE for deterministic baselines). RAPSD analysis shows improved retention of high-frequency variance relative to deep learning baselines while avoiding the texture-only limitations of Optical Flow. Spatial maps demonstrate that our diffusion model significantly outperforms the baselines, indicating smaller errors over the study region. Two case studies show coherent structures in Tb forecasts, sharper gradients, and improved localization of evolving cold features. Overall, coupling diffusion with 3D U-Net delivers more structurally faithful satellite nowcasts than traditional extrapolation and deep learning baselines, particularly at short to intermediate leads.

**Supplementary Information:**

The online version contains supplementary material available at 10.1038/s41598-025-34207-9.

## Introduction

Geostationary weather satellite imagers provide continuous passive infrared (IR) sensed information, critical for near-real-time and short-term forecasts of weather-scale phenomena. These sensors, such as Spinning Enhanced Visible and InfraRed Imager (SEVIRI) on Meteosat, Advanced Baseline Imager (ABI) on GOES-R series, and the Himawari Advanced Himawari Imager (AHI) series, track cloud-top temperatures and motion, offering broad coverage, which is especially valuable in regions with sparse ground-based networks. These timely satellite observations are critical for monitoring rapidly developing convective weather systems, which play an important role in precipitation, especially in semiarid and arid areas. They are equally valuable for tracking large-scale stratiform and frontal cloud systems that influence regional water availability and radiation balance. Furthermore, they provide important input data for nowcasting, short-term (0–6 h) forecasting, of evolving cloud systems, which is vital for early warnings of storms, rainfall, aviation hazards, and precipitation enhancement planning. Traditionally, forecasters relied on extrapolating cloud motions (e.g., Optical Flow techniques) and conceptual rules to project current satellite observations into the near future^1,2^. However, such methods struggle with complex cloud growth/decay and lead-time limitations. Convective evolution’s inherently chaotic, multiscale nature makes accurate short-term prediction very challenging. Coarse resolution numerical weather prediction (NWP) models face a spin-up problem and high computational cost at nowcasting scales, often failing to track small-scale storms in the first 1–2 h^3^. This gap motivates data-driven, observational-based nowcasting approaches that can rapidly learn patterns from satellite image sequences to improve forecast timeliness and accuracy.

Recent advancements in machine learning have enhanced satellite-based cloud retrievals and nowcasting. Recurrent neural networks (RNNs) such as convolutional long short-term memory (ConvLSTM) networks and Trajectory GRU (TrajGRU) have demonstrated the capability to capture cloud evolution dynamics by modeling satellite image sequences as video data, significantly surpassing traditional static extrapolation methods^4,5^. Berthomier et al. 2020^6^ applied a U-Net convolutional neural network (CNN)^7^ to Meteosat Second Generation (MSG) imagery, achieving more accurate cloud cover nowcasting, with 15 min temporal resolution, up to 90 min ahead compared to RNN, conventional Optical Flow, and physical benchmarks. Lagerquist et al. 2021^8^ similarly utilized a CNN-based U-Net on Himawari-8 multi-channel brightness temperature (Tb) imagery to predict thunderstorm occurrence up to two hours in advance, demonstrating comparable accuracy to radar-based methods in regions lacking dense radar coverage. Recent experiments have also shown that transformers effectively learn complex relationships in satellite imagery. Küçük et al. 2024^9^ showed that transformer models utilizing multispectral IR data alongside lightning observations significantly improve the realism of predicted radar reflectivity fields, capturing detailed convective structures.

Despite these successes, limitations remain in satellite cloud nowcasting methods. Optical Flow techniques, widely used in operational settings (due to their simplicity), are unable to nowcast cloud formation or dissipation processes, relying solely on extrapolating current motion vectors. This limits the skillful forecast window typically to 1–2 h^10^. Recurrent models often suffer from error accumulation over long sequences, while CNN-based architectures (e.g., U-Net) and ConvLSTM often show initial skill improvements but tend to produce increasingly blurry forecasts as lead time grows, a consequence of averaging effects inherent in pixelwise loss functions that effectively smooth uncertain future states^11,12^. Generative adversarial networks (GANs) and transformers have been explored to mitigate these limitations, but GANs frequently encounter training instabilities such as mode collapse, and transformers require substantial computational resources and large training datasets to generalize effectively^13–16^.

Generative AI-based nowcasting can tackle the shortcomings of conventional models by reframing forecasting as a learned, image-generation task^17,18^. In particular, diffusion models cast the nowcasting problem as the reverse of a noising process, allowing them to model the full distribution of plausible future cloud states, including formation and dissipation, rather than simply extrapolating existing motion vectors or averaging over uncertain outcomes^19^. This feature makes diffusion models a powerful alternative to GANs and transformers for high-fidelity^20^. Diffusion models are known for extremely high-quality image synthesis and have the advantage of stable training (no mode collapse) at the cost of slow sampling. Early implementations such as DiffusionSat^21^ have demonstrated superiority and detail preservation in generated satellite imagery compared to conventional deep learning models, highlighting diffusion models’ capability to address the complex spatiotemporal dynamics inherent in cloud evolution. Nai et al. 20,204^22^, Dai et al. 2024^23^, and Chen et al. 2025^24^ further validated these benefits, reporting diffusion-based nowcasting models that maintain forecast quality and sharpness over extended lead times, significantly alleviating common issues of blurriness and structural uncertainty prevalent in other well-known frameworks such as the NowcastNet^14^ and pySTEP^25^.

Our study explores a novel framework that integrates a denoising diffusion probabilistic model with a 3D U-Net architecture over the predominantly arid United Arab Emirates (UAE) and its neighboring region to address several fundamental limitations in satellite-based nowcasting, degraded spatial sharpness at longer lead times, and limited predictive horizons. By leveraging the generative capabilities of diffusion models, the proposed approach generates deterministic nowcasts from a set of probabilistic forecasts that preserve fine-scale spatial features while extending temporal coherence across forecast frames. In contrast to CNNs and RNNs, this architecture simultaneously encodes spatial and temporal dependencies through volumetric convolutions applied to sequences of geostationary IR observations. While diffusion-based models have recently been explored in satellite-based retrievals, integrating a diffusion model with a 3D U-Net for high spatial and temporal resolution geostationary IR data is the cutting-edge of nowcasting research^26–31^. In this paper, we are focusing on applying the diffusion models to a geostationary satellite that provides coverage over the UAE as a proof of concept. The selection of the study domain is motivated by cloud‑seeding efforts in this region that typically require accurate, high‑resolution forecasts of cloud‑top evolution to target seeding locations and for related flight planning operations^32–34^. Convective systems contribute substantially to the annual precipitation amount in the predominantly arid UAE region^35,36^. Moreover, the characteristics of the convective clouds are critical for identifying cloud-seeding targets^37^. Consequently, timely nowcasting of cloud characteristics is in high demand for cloud seeding and precipitation enhancement efforts in the UAE and the surrounding region. Previous conventional nowcasting tends to smooth out the fine‑scale updraft pockets and supercooled cloud regions where seeding agents are most effective.

Here we integrate a denoising diffusion probabilistic model with a 3D U-Net to generate six-hour IR Tb nowcasts from geostationary imagery and evaluate performance over the UAE region using an independent test period. We form a nine-member diffusion ensemble and use the mean as a deterministic forecast, benchmark against a 3D U-Net, a ConvLSTM, and a dense-optical-flow method, and assess skill with multiple statistical metrics.

## Data and methodology

### Satellite data

**Fig. 1:**
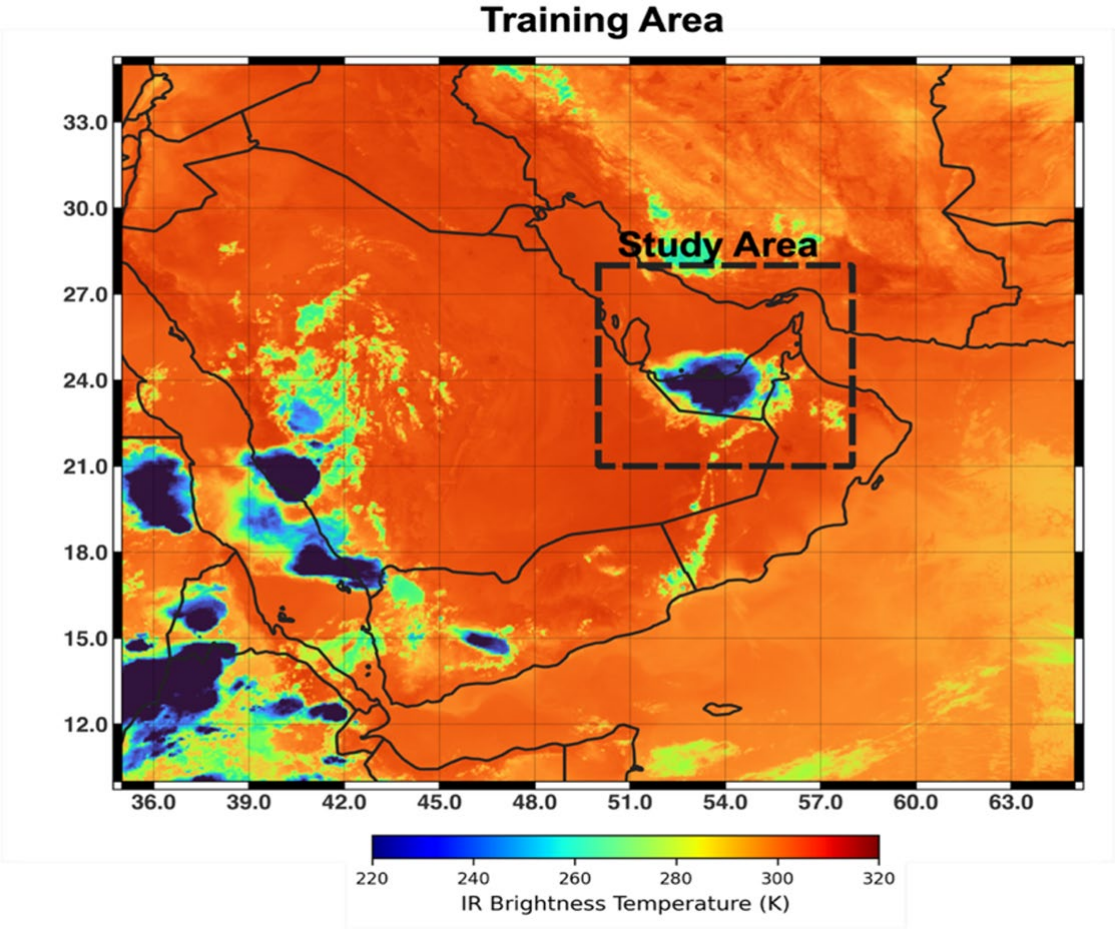
Training and test domains, overlaid on a sample IR Tb image from 18 August 2019. The outer domain (solid boundary) represents the region used for model training, while the inner dashed box delineates the test area covering 21°–28° N, 50°–58° E.

We use near-real-time level 1.5 IR Tb at ~ 10.8 μm channel from Spinning Enhanced Visible and Infrared Imager (SEVIRI) sensor onboard the METEOSAT Second Generation (MSG) satellites with Indian Ocean Data Coverage (IODC). This longwave IR channel is selected because it serves as a standard thermal infrared window channel across most geostationary satellites globally (e.g., GOES-R, Himawari), enabling easier merging and harmonization of datasets from different platforms^38^. The 10.8 μm channel known as “clean” IR channel is less affected by atmospheric water vapor absorption and more sensitive to cloud-top height and temperature than other IR window bands, enabling accurate monitoring of cloud evolution and radiative cooling and warming processes that are critical for short-term forecasting. The 15-minute clean longwave IR information from 2017 to 2022 used in our investigations is geolocated to a uniform 3-km grid, resulting in images containing calibrated, radiance linearized, and Earth-located information appropriate for cloud nowcasting. The study area covers latitudes 21°-28° N and longitudes 50°-58° E, which covers the UAE (Fig. 1), while for model training, we used a larger domain encompassing the Arabian Peninsula (latitudes 10°-35° N and longitudes 35°-65° E) to substantially expand our training dataset. All IR Tbs data are mapped into the Coordinate Reference System EPSG:4326 (WGS 84) using the native sensor geometry and nearest-neighbor geolocation mapping producing a training-domain array of approximately 835 × 1000 pixels. All 15-minute timestamps (00:00–23:59 UTC) during July–September of 2017–2022 are used, except for frames with more than 1% missing pixels. We selected the warm season (July, August, September) for cloud nowcasting due to the unique meteorological conditions prevalent over the study area^36,39^. This period is characterized by intense solar heating, triggering convection and towering cumulus development, while also including widespread non-convective clouds and mesoscale systems important for satellite-based forecasting^40^.

### 3D U-Net diffusion model

**Fig. 2:**
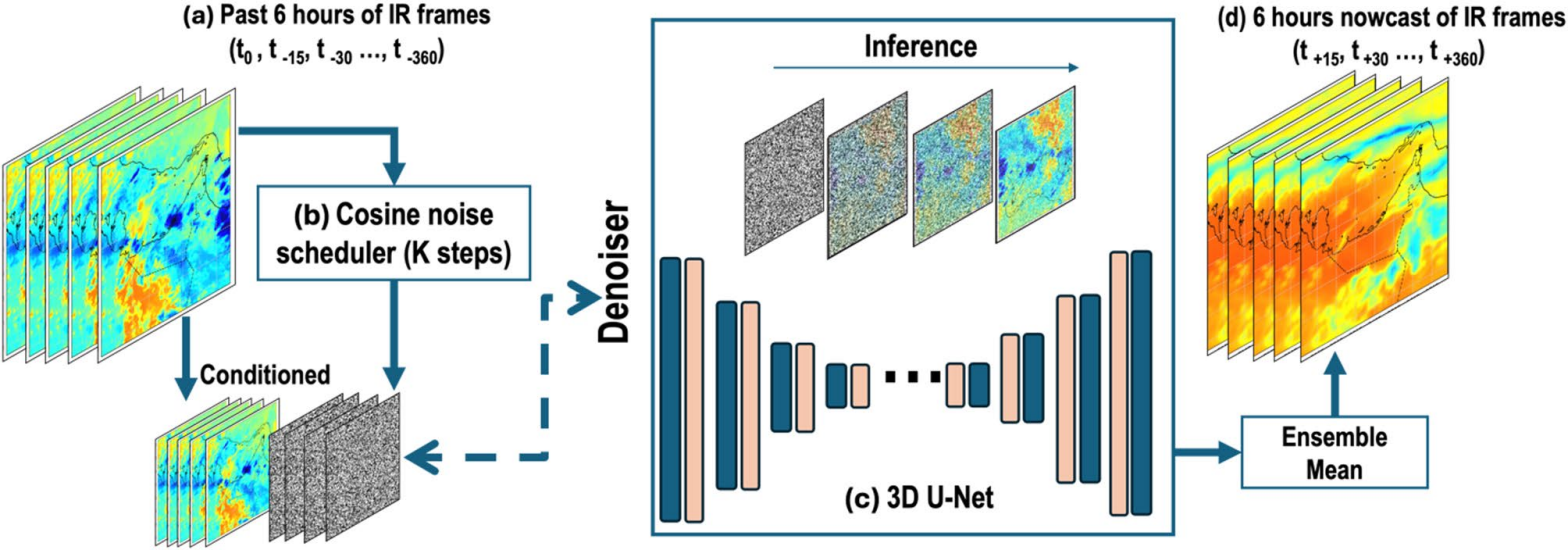
Schematic of the conditional 3D U-Net Diffusion model. (a) Six hours of input infrared brightness temperatures (IR Tbs) are used to nowcast (d) the next six hours of IR Tbs, guided by (b) a cosine scheduler and (c) a 3D U-Net denoiser.

Diffusion models are a class of generative models that have recently achieved state-of-the-art results in image and audio generation^43^. They have been applied to various applications such as text-to-image^44^, text-to-video^45^, and video-to-video^46^ by appropriate conditioning. Melnik et al. 2024^47^ reviewed video diffusion models and their applications. The U-Net is the most widely used architecture for the denoising steps in diffusion models^47–49^. The flexibility in conditioning means diffusion U-Nets act as a general solution for many tasks: by providing part of an image or a textual description, one can generate coherent outputs matching the condition *Z*. In the stochastic forward process (as explained in detail in Ho et al. 2020^50^), starting from a real image ${X_0}$, the model adds a small amount of Gaussian noise at each time step for *k = 1*,* 2*,* .*,* K* (total number of diffusion iterations is *K*) until we obtain pure noise ${X_K} \approx N\left( {{X_K},~0,I} \right)$. We can define this process as:

1 $$p({X_k}|{X_{k - 1}})=N\left( {{X_k};~\sqrt {1 - {\beta _k}} {X_{k - 1}},~{\beta _k}I} \right)$$

where β_1_, …, β_K_ control the variance of added noise (0 < β_k_ < 1) and is controlled by the noise scheduler (as shown in Fig. 2). In the stochastic reverse process, the model learns a reverse diffusion process that can gradually convert noise into a coherent sample by inverting a forward process conditioned on Z information (here, initial IR frames), as the model uses this information when producing samples:

2 $$p_{\theta } (X_{{k - 1}} |X_{k} ,Z~) = \frac{{p(X_{k} |X_{{k - 1}} )~p(X_{{k - 1}} |Z)}}{{p(X_{k} |Z)}}$$

A neural network (e.g., U-Net^7^ with parameters θ takes the noisy image in iteration k ${X_k}$, with the conditioning information Z, to estimate the expected value of ${X_{k - 1}}$. During training, a diffusion model simulates a Markovian noising process, then the model learns optimal θ to denoise, given a noisy image at an arbitrary step. By training on a weighted combination of these denoising objectives across all timesteps, the model approximates the reverse of the diffusion chain. Training minimizes the MAE between the true noise ɛ and the network’s prediction ɛ_θ_ (X_k_, k, Z):

3 $$L~=~{E_{{X_0},\varepsilon ,k~}}\left[ {\left| {\varepsilon - {\varepsilon _\theta }\left( {{X_k},k,Z} \right)} \right|} \right],~~~$$

$$where~{X_k}=\sqrt {\underline {{{\alpha _k}}} } \,{X_0}+\sqrt {1 - \underline {{{\alpha _k}}} } \varepsilon ~and~\underline {{{\alpha _k}}} =\mathop \prod \limits_{{i=1}}^{k} \left( {1 - {\beta _i}} \right).$$

We refer to Ho et al. 2022^51^ for a precise description of extended diffusion models for video generation by using 3D U-Net architectures by Çiçek et al. 2016^41^ as the backbone for the denoiser network in the model. In this design, the model processes a whole chunk of image frames as a single data sample (generating future frames given a few initial frames), and the 3D U-Net captures dependencies along the time dimension as well as across spatial dimensions, using 3D convolutions and attention mechanisms. Common strategies in this approach include factorized attention (applying self-attention within frames and across frames separately) and relative positional embeddings for time to help the model understand frame ordering. In a conditional image-to-image diffusion model, the condition is the set of initial frames (i.e., history frames), and the model learns to generate future frames stochastically.

In this study, we implemented the Ho et al. 2022^51^ video diffusion model with a modified 3D U-Net architecture integrated within a denoising diffusion framework (hereafter 3D U-Net Diffusion) for nowcasting sequential IR frames conditioned on initial IR inputs (Fig. 2). The denoiser model, shown as (c) in Fig. 2, includes four encoders, one bottleneck, and four decoder stages. The base dimension of the feature maps is set to 256 and progressively increased by multiples of two, yielding dimensions of 256, 512, 1024, and 2048 at successive encoder stages. Each encoder stage employs residual blocks using 3D convolutions (Two 3D convolutions with group normalization and the SiLU activation function with feature-wise linear modulation, conditioned by the sinusoidal time embedding to capture local spatiotemporal features). Spatial features within individual frames are extracted using convolutional kernels sized 1 × 7 × 7, followed by spatial linear attention for global spatial context and temporal attention for interframe dependencies and spatial down-sampling via 1 × 4 × 4 convolutions. Temporal attention mechanisms are designed to model interframe relationships at full spatial resolution. The temporal self-attention is multi-head attention applied along the frame axis at every location (pixel), and rotary positional encodings mark frame order. Each pixel sees its past and future context early, improving motion awareness.

The bottleneck is one residual block, a complete spatial self-attention module, and a full temporal self-attention module, enabling comprehensive integration of global spatiotemporal contexts at the lowest spatial dimension. The decoder mirrors the encoder’s architecture, progressively reconstructing the output by concatenating encoder skip-connections at corresponding resolutions. Each decoder stage similarly employs two residual blocks, spatial linear attention, temporal attention, and spatial upsampling through trilinear interpolation (to avoid deconvolution artifacts). The final convolutional output layer produces predictions matching the input spatial resolution and temporal length. The diffusion process incorporates a cosine noise schedule (shown as (b) in Fig. 2) over *K* = 100 timesteps and is conditioned explicitly on initial frames, employing an MAE loss focused on denoising future frames. The cosine noise schedule^52^ follows a cosine curve as a function of timestep (starting near zero and approaching one at the final step). This strategy tends to allocate more noise to later timesteps while preserving some signal in earlier ones, often leading to improved sample quality over a linear schedule.

Our 3D U‑Net Diffusion model contains approximately 520 million parameters, blending local 3D convolutional dynamics with dual (spatial and temporal) attention at every level. The stochastic denoising process yields high‑detail, realistic forecasts of future infrared (IR) frames. For each input history (initial IR frames), we generate nine forecast ensembles with random noise seeds making an ensemble of nine; the ensemble mean serves as our deterministic forecast for evaluation. We train and validate on data from 2017 to 2021 and reserve 2022 as an independent test dataset, ensuring rigorous out‑of‑sample assessment. Within the 2017–2021 period, we allocate 80% of the samples for training and 20% for validation, using a time-ordered split to prevent temporal leakage. All input–output sequences (history and future frames) must fall entirely within their assigned date range, and no sequence is permitted to cross between splits, ensuring that identical timestamps never appear in both training and validation sets. We construct input–output sequences at 3 km spatial resolution consisting of 24 historical frames (past 6 h) and 24 prediction frames (next 6 h), each sampled at 15-minute intervals. IR Tb values are normalized using channel statistics computed over the training period to ensure stable and balanced model optimization. Only contiguous sequences containing all required history and future frames are retained. Thus, the full spatiotemporal input and output tensors are [24 × 835 × 1000] and [24 × 835 × 1000], ] respectively.

### Benchmark models

This study uses the 3D U-Net^41^ for spatiotemporal IR Tb nowcasting as a benchmark. Using 3‑D convolutions allows the network to exploit spatial structure and short‑term temporal trends simultaneously. Explicitly providing the difference channel supplies a local estimate of the first temporal derivative, which the model leverages to sharpen motion extrapolation. The network ingests a volume of the 24 most recent IR images (last 6-hour IR data with 15-minute time intervals) together with their first‑order temporal differences (two input channels) and predicts the subsequent 24 IR frames (one output channel). This configuration is similar to the 3D U-Net architecture used in the 3D U-Net Diffusion model (section 3D U-Net diffusion model) and is trained with a masked mean-absolute-error (MAE) loss function. The model masks the loss at any invalid sample (e.g., missing values are flagged as -1000 value). This ensures that invalid data do not contribute to the gradient. Optimization employs AdamW^42^; learning rate = 1 × 10^–4^, weight decay = 1 × 10^–4^ with gradient clipping limited to a maximum value of 10 ($\left\| g \right\|_{2}$ ≤ 10, the notation $\left\| g \right\|_{2}$refers to the Euclidean (L2) norm of the gradient vector g), and up to 100 epochs with early stopping (patience of 20 epochs) using PyTorch DataParallel over up to four NVIDIA A100 GPUs.

We trained a four-layer ConvLSTM with 64 hidden channels per layer and convolutional kernels for all gates and state transitions as another benchmark^4^. We used the same training data and strategy as the 3D U-Net and 3D U-Net Diffusion models to ensure a fair comparison. Gates follow the standard LSTM formulation, input, forget, and output with sigmoid activations, while the cell update uses tanh. Convolutions replace affine transforms, so that spatial locality is preserved (refer to study by Shi et al. 2015^4^ for detailed description). Given an input sequence of 24 IR frames, the network encodes spatiotemporal dependencies and predicts 24 future frames at the 3 km spatial resolution. Training minimizes masked MAE over valid pixels only (invalid data are excluded via the same mask used for the 3D U-Net and the diffusion models) using Adam optimizer with early stopping strategy.

In addition to 3D U-Net and ConvLSTM baselines models in this study, Farneback dense Optical Flow method (hereafter Optical Flow) is also implemented on consecutive IR frames to extract the advection flows for each pixel (refer to study by Akbari Asanjan et al. 2018^53^ for detailed description). Flow fields are computed on a 3-level pyramid (scale 0.5), with a 15 × 15 neighborhood window, three iterations per level, and a polynomial expansion (degree 5, smoothing 1.2). Smoothing 1.2 parameter in the Farneback polynomial expansion refers only to local gradient smoothing within the Optical Flow estimation and does not introduce any temporal smoothing across forecast lead times. To reduce pairwise noise and reflect recent motion, we estimate per-pixel flow over the last six consecutive input pairs and take the arithmetic mean of the resulting vectors at each pixel. This yields a robust, recent motion field that assumes steady advection forward at 15-min increments. This design preserves fine-scale texture but does not artificially reduce error metrics at longer leads. As a result, the method behaves as a pure advection forecast that maintains small-scale variance rather than regressing toward a smoother mean field.

### Evaluation metrics

The performance of deterministic nowcasting models is evaluated with bias (lower is better and the ideal value is 0) and centered root-mean-square error (CRMSE; lower is better and the ideal value is 0):

4 $${\mathrm{Bias}}={{\mathrm{P}}_{\mathrm{i}}} - {{\mathrm{O}}_{\mathrm{i}}}{\mathrm{~}}$$

5 $${\mathrm{CRMSE}} = \sqrt {\frac{1}{n}\mathop \sum \limits_{{i = 1}}^{n} \left( {(P_{i} - \bar{P}) - (O_{i} - \bar{O})} \right)^{2} }$$

Where P_i_ represents the *i*th forecasts, O_i_ is the corresponding observation, $\bar {P}$ refers to the mean of predictions, and $\bar {O}$ is the mean of observations, with *n* denoting the total number of samples. In addition, we used the Pearson correlation coefficient (hereafter correlation; higher is better, and the perfect value is 1) as:

6 $${\text{Correlation = }}\frac{1}{n}\frac{{\mathop \sum \nolimits_{{i = 1}}^{n} \left( {P_{i} - \bar{P}} \right)\left( {O_{i} - \bar{O}} \right)}}{{\sqrt {\mathop \sum \nolimits_{{i = 1}}^{n} \left( {P_{i} - \bar{P}} \right)^{2} } \sqrt {\mathop \sum \nolimits_{{i = 1}}^{n} \left( {O_{i} - \bar{O}} \right)^{2} } }}$$

The critical success index (CSI) is considered for categorical assessments. CSI evaluates how well each model detects cold-Tb clouds compared with observations and is defined as:

7 $${\mathrm{CSI}}=\frac{{\mathrm{H}}}{{{\mathrm{H}}+{\mathrm{F}}+{\mathrm{M}}}}$$

An event is defined when Tb falls below 275 K. Accordingly, H (hit) indicates that both the model and reference observation detect the event (Tb < 275 K), M (miss) identifies an observed event that the model fails to predict, and F (false alarm) indicates cold-Tb cloud regions predicted by the model but absent in the observation. We use a 275 K threshold, consistent with common practice in IR-based cloud detection^54,55^, because it captures not only deep convective systems but also warm-rain clouds and overlapping cloud layers that can have Tb warmer than deep-convection thresholds (240–250 K). This choice is particularly important in the UAE region, where many precipitating and evolving multi-layer cloud systems exhibit moderately cold Tb that would be missed when using colder thresholds.

To evaluate the probabilistic nowcasts from 3D U-Net Diffusion, CRPS (Continuous Ranked Probability Score) is used, which measures the difference between the predicted cumulative distribution function (CDF) and the observed value. For a forecast CDF F and an observation O:

8 $${\mathrm{CRPS~}}\left( {{\mathrm{F}},{\mathrm{~O}}} \right)=\mathop \smallint \limits_{{ - \infty }}^{\infty } {\left[ {F\left( P \right) - {\mathrm{~}}1{\mathrm{~}}\left( {{\mathrm{P}} \geqslant {\mathrm{O}}} \right)} \right]^2}~dP~$$

where $1{\mathrm{~}}\left( {{\mathrm{P}} \geqslant {\mathrm{O}}} \right)$ is the indicator function (1 if ${\mathrm{P}} \geqslant {\mathrm{O}}$, 0 otherwise). For deterministic nowcasts, CRPS generates mean absolute error (MAE):

9 $${\mathrm{MAE}}=\frac{1}{{\mathrm{n}}}\mathop \sum \limits_{{{\mathrm{i}}=1}}^{{\mathrm{n}}} \left| {{{\mathrm{P}}_{\mathrm{i}}} - {{\mathrm{O}}_{\mathrm{i}}}} \right|$$

To quantify structural fidelity across spatial scales, we compute the radially averaged power spectral density (RAPSD) of Tb fields. For each forecast (and the corresponding observation), the 2D field is converted to the frequency domain via a 2D discrete Fourier transform; the power spectrum shows how much variance (or “energy”) is contained at each frequency. Power is then radially averaged over concentric annuli centered at the origin of the frequency plane to obtain a one-dimensional spectrum of mean power versus spatial frequency (cycles per pixel)^56,57^.

Finally, we include the Structural Similarity Index (SSIM) as an additional evaluation metric. This is a perceptual measure designed to quantify the similarity of Tb predictions (P) to the observation (O). Rather than comparing individual pixel values directly, SSIM evaluates three complementary aspects of image quality: structural similarity, luminance, and contrast^58^. These are combined into a single score defined as:

10 $$SSIM=\frac{{\left( {2{\mu _P}{\mu _O}+~{C_1}} \right)\left( {2{\sigma _{PO}}+~{C_2}} \right)}}{{\left( {\mu _{P}^{2}+\mu _{O}^{2}+{C_1}} \right)~\left( {\sigma _{P}^{2}+~\sigma _{O}^{2}+{C_2}} \right)}}$$

where ${{\mathrm{\boldsymbol{\upmu}}}_{\mathrm{P}}}$and ${{\mathrm{\boldsymbol{\upmu}}}_{\mathrm{O}}}$are the mean values of the predicted and observed Tb fields, ${\mathrm{\boldsymbol{\upsigma}}}_{{\mathrm{P}}}^{2}$ and ${\mathrm{\boldsymbol{\upsigma}}}_{{\mathrm{O}}}^{2}~$their corresponding variances. ${{\mathrm{\boldsymbol{\upsigma}}}_{{\mathrm{PO}}}}$ is the covariance between observation and prediction. ${{\mathrm{C}}_1}$ and$~{{\mathrm{C}}_2}$ are small constants that stabilize the division and reduce exaggerated sensitivity when the local means or variances are very small:

11 $${C_{1=~{{\left( {L~{K_1}} \right)}^2}}}~~and~~{C_{2=~{{\left( {L~{K_2}} \right)}^2}}}$$

where L is the dynamic range of the observation, with K1 = 0.01 and K2 = 0.03. SSIM ranges from − 1 to 1, with 1 indicating perfect structural agreement. Because it emphasizes preservation of spatial patterns and edges, SSIM provides a perceptually meaningful complement to traditional metrics^59^, highlighting visual degradations such as blurriness that are especially relevant for evaluating nowcasts of Tbs.

## Results and discussions

**Fig. 3:**
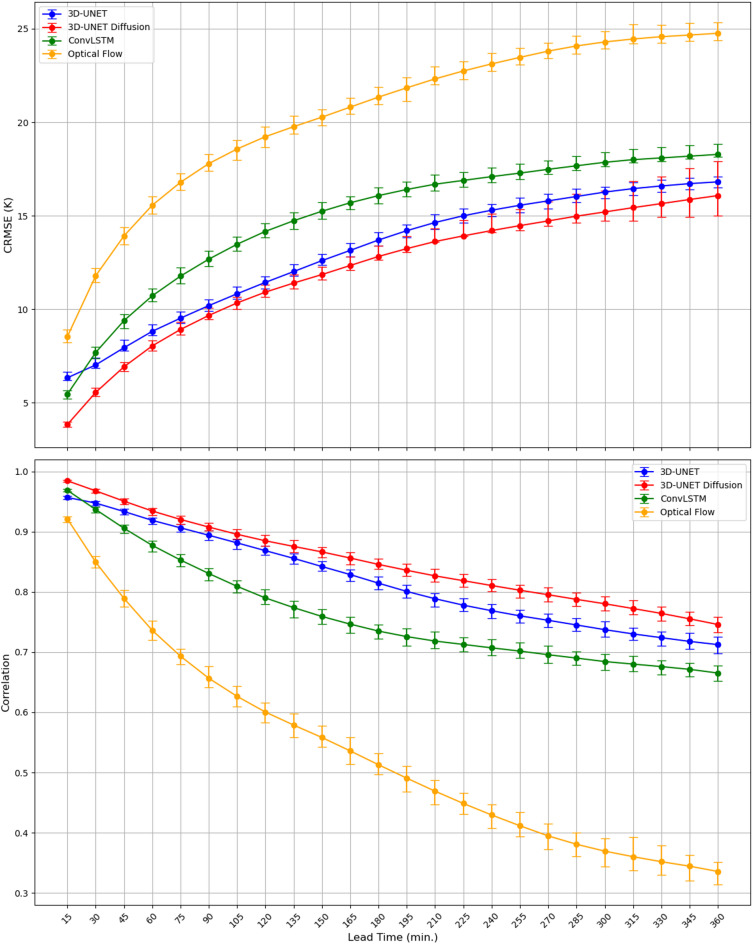
Centered root mean square error (CRMSE; top) and Pearson correlation coefficient (Correlation; bottom) as a function of lead time for the 3D U-Net model (blue) and 3D U-Net Diffusion (red), ConvLSTM (green) and Optical Flow (yellow) with 95% confidence intervals from 1000 bootstrapping.

This section compares the performance of 3D U-Net Diffusion model (ensemble mean) compared to 3D U-Net, ConvLSTM and Optical Flow benchmarks. This comparison is across the study domain over the entire independent test period (July—September 2022) considering all forecast lead times (15 min to 6 h at 15-minute intervals, initialized every 15 min). As shown in the top panel of Fig. 3, the CRMSE for all nowcasting models increases with lead time, reflecting the expected degradation of forecast skill over time. At the first lead time (15-min), the CRMSE is approximately 2.5 K for the 3D U-Net Diffusion model, compared to ~ 6 K for the 3D U-Net model, ~ 5.5 K for ConvLSTM, and ~ 8 K for Optical Flow. While these values increase to about 8 K, 9 K, 11 K, and 15.5 K for 3D U-Net Diffusion, 3D U-Net, ConvLSTM and Optical Flow, respectively at 1-hour lead time. This highlights the diffusion models’ superior accuracy at short lead times. The gap between 3D U-Net Diffusion, and 3D U-Net models narrows as lead time increases to 135 min, while Optical Flow and ConvLSTM degrade rapidly, which is expected, given the tendency of predictions to become increasingly blurry over time. The diffusion consistently outperforms 3D U-Net across all lead times though Optical Flow maintains the largest errors throughout. Part of this behavior reflects the fact that our Farneback configuration advects textures without applying temporal smoothing. While smoothing can reduce RMSE and increase correlation by damping variance, it also suppresses high-frequency cloud structures. Our advection configuration preserves these finer-scale features, which can increase CRMSE but avoids producing artificially smooth fields. By 360 min (six hours) lead time, the CRMSE of 3D U-Net and diffusion converge near 16–17 K, whereas ConvLSTM remains higher (~ 18 K) and Optical Flow exceeds 24 K. The 95% confidence intervals (error bars in Fig. 3) show narrower spread at early lead times and wider intervals at later horizons, especially for ConvLSTM and Optical Flow.

Correlation analysis (bottom panel of Fig. 3) confirmed these findings, with the diffusion model maintaining higher correlation values (above 0.88) through the first two hours followed by U-Net, ConvLSTM, and Optical Flow. As lead time increases, correlations decline for all models, dropping more sharply for Optical Flow, which falls below 0.5 after ~ 180 min. The gap between the models’ correlation values widens as lead time increases, while the 3D U-Net Diffusion model shows higher correlation values at all lead times when compared with other baseline models, indicating better preservation of the spatiotemporal structure of the observed and predicted IR Tbs. At 360-min lead time, correlations for both models drop below 0.75 for diffusion, 0.72 for U-Net, 0.7 for ConvLSTM, and below 0.4 for Optical Flow. These comparisons confirm that while all models experience skill degradation with increasing lead time, the diffusion model consistently maintains higher deterministic accuracy and higher correlations, demonstrating its robustness relative to both deep learning and traditional baselines.

**Fig. 4:**
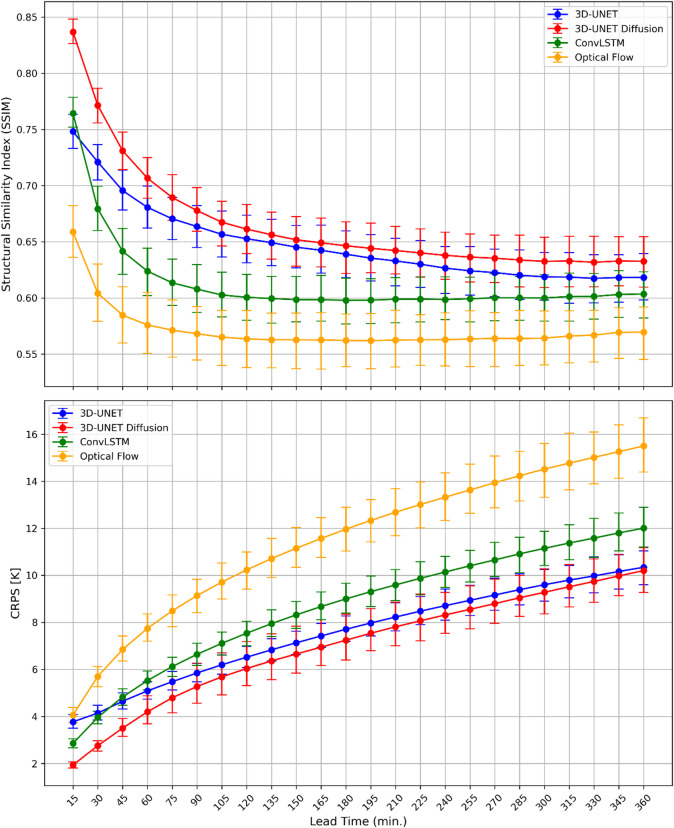
Structural Similarity Index (SSIM, top) and Continuous Ranked Probability Score (CRPS, bottom) as a function of lead time for 3D U-Net model (blue) and 3D U-Net Diffusion (red), ConvLSTM (green) and Optical Flow (yellow) with 95% confidence intervals from 1000 bootstrapping.

To further evaluate the diffusion model beyond conventional statistics, we examine two complementary metrics SSIM, which measures perceptual quality, and the CRPS which assesses probabilistic skill (Fig. 4). As shown in Figs. 3D and 4 U-Net Diffusion model achieves the highest SSIM values at all lead times, starting near 0.84 at 15 min and maintaining values above 0.65 before 6 h lead time. This indicates superior preservation of structural details compared to the other methods. The 3D U-Net performs second-best in all the lead times except first 15 min (ConvLSTM outperforms 3D U-Net) converging to ~ 0.62 by 6 h. ConvLSTM exhibits noticeably lower scores compared to 3D U-Net and diffusion models, and its SSIM declines more quickly than 3D U-Net model. While the SSIM values for ConvLSTM stabilize near 0.60 after the first 90 min, the Optical Flow degrades the most, dropping below 0.58 within the first hour and remaining low thereafter. These results highlight that the diffusion framework reduces blurriness and maintains sharper, more perceptually realistic cloud-top structures across extended lead times.

Although the main focus of this study is deterministic nowcasting, we added CRPS analysis to provide an additional perspective on diffusion model skill, as a complement to the CRMSE and correlation analyses presented earlier. CRPS values (bottom panel of Fig. 4) are calculated for 9 generated ensembles from 3D U-Net Diffusion, as well as for the deterministic forecasts from 3D U-Net, ConvLSTM, and Optical Flow, for which CRPS generates MAE. The results show that the diffusion model achieves the lowest CRPS, approximately 2 K, at the shortest lead time (15 min), increasing gradually to ~ 10 K by 6 h. In comparison, 3D U-Net and Optical Flow begin around 4 K, and ConvLSTM around 3 K, with errors growing to ~ 10 K for U-Net, ~ 12 K for ConvLSTM, and over 15 K for Optical Flow at 6 h. Across all lead times, the diffusion model maintains the lowest CRPS values, demonstrating reduced forecast error and improved accuracy relative to both deep learning and traditional benchmarks. These results confirm that the ensembles from the 3D U-Net Diffusion model yield more accurate Tb forecasts and that CRPS is a valuable complement to the CRMSE plots by summarizing distributional accuracy (and MAE for deterministic models) across lead times, while CRMSE clarifies how offsets and error variance contribute to total accuracy. A more comprehensive probabilistic evaluation is planned for future work, as it falls outside the scope of this deterministic-focused study.

**Fig. 5:**
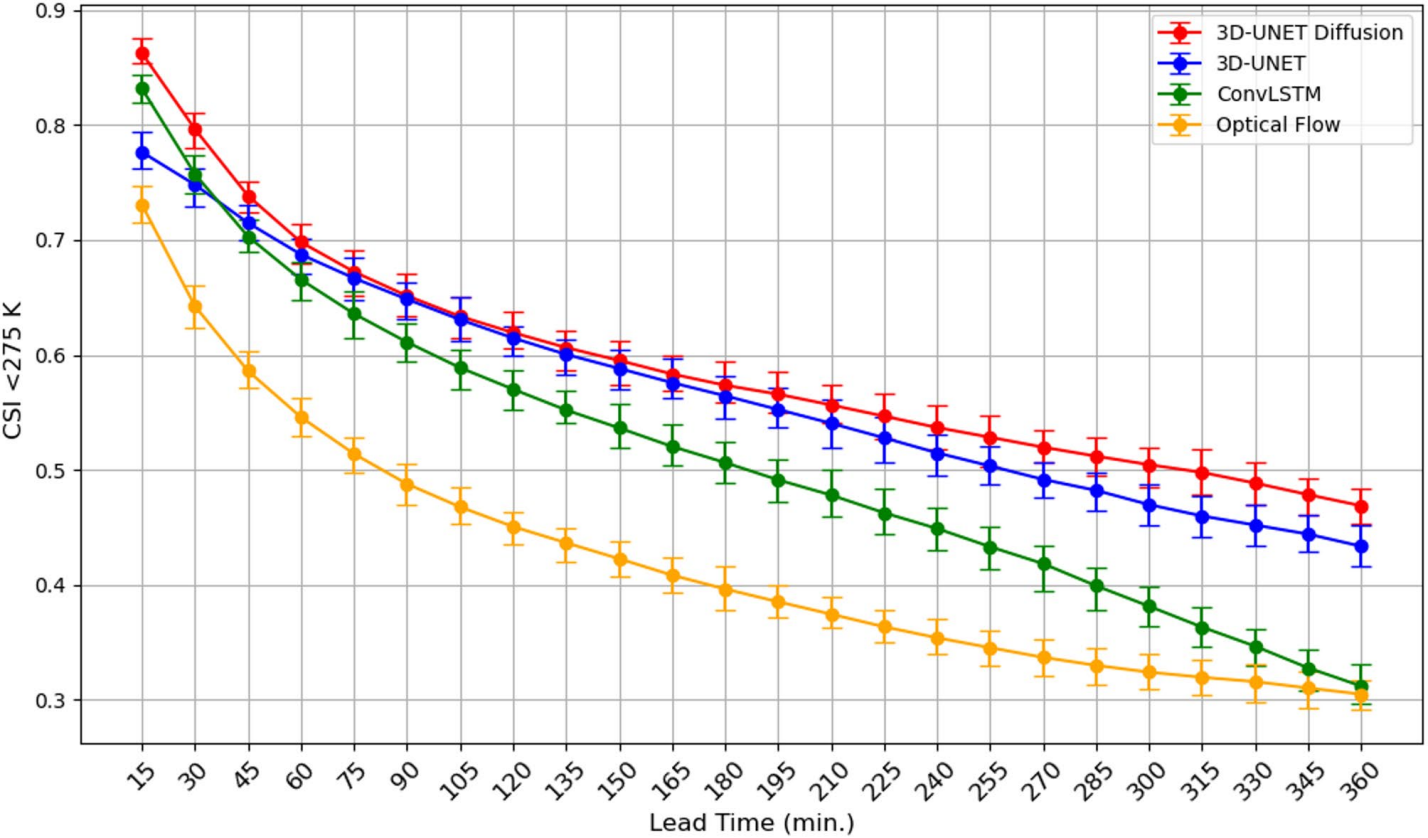
Critical success index (CSI) for brightness temperatures (Tbs) less than 275 K as a function of lead time for 3D U-Net model (blue) and 3D U-Net Diffusion (red), ConvLSTM (green) and Optical Flow (yellow) with 95% confidence intervals from 1000 bootstrapping.

Beyond continuous error statistics, we evaluate categorical forecast skill using the CSI to quantify the ability of each model to detect cold-Tb cloud regions (Tb < 275 K). Figure 5 shows the CSI as a function of lead time. At the shortest lead time (15 min), the 3D U-Net Diffusion model achieves the highest CSI (~ 0.88), followed by ConvLSTM (~ 0.84), 3D U-Net (~ 0.78), and Optical Flow (~ 0.73). CSI declines steadily with lead time for all models, reaching values below 0.6 by 180 min as accurately predicting the colder portions of evolving Tb fields becomes increasingly difficult. Over this period, diffusion and U-Net maintain comparable skill, while ConvLSTM degrades earlier and Optical Flow deteriorates most rapidly. At longer horizons (beyond 3 h), all models converge toward lower CSI values, but diffusion consistently preserves a small yet systematic advantage. The improved CSI of the diffusion model reflects its enhanced ability to preserve fine-scale cold-Tb cloud structures and maintain spatial coherence, although, similar to other approaches, this sharpness advantage diminishes at extended lead times due to increasing smoothing of cloud-top features.

**Fig. 6:**
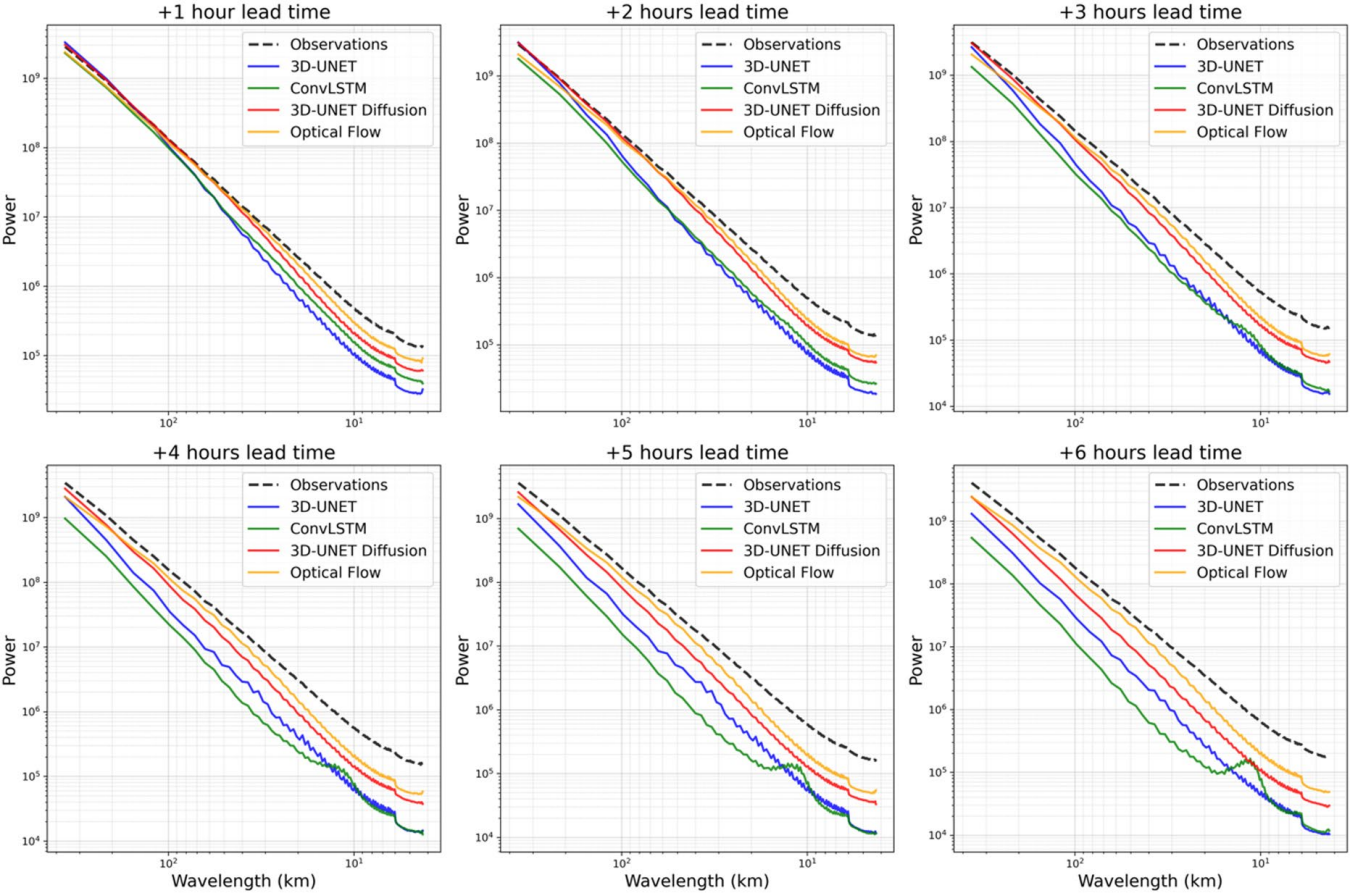
Radially averaged power spectral density (RAPSD) of observed and forecasted Tbs for 1–6 h lead times (a–f). Spectra are averaged over the test period (July–September 2022) for regions with Tbs less than 275 K. The black dashed line shows observations, while red, blue, green, and yellow lines represent 3D U-Net Diffusion, 3D U-Net, ConvLSTM, and Optical Flow forecasts, respectively.

To complement the categorical assessment of cold cloud detection and the structural evaluation provided by SSIM, we examine the Radially Averaged Power Spectral Density (RAPSD) to assess scale-dependent smoothing (Fig. 6). Each panel provides a scale-dependent diagnostic of forecast performance by quantifying how variance in Tbs is distributed across spatial frequencies at lead times of 1 to 6 h. Alignment between model and observed spectra indicates structural fidelity, whereas deficits, particularly at high frequencies, show the extent of smoothing or blurring introduced by a model. High frequencies (small wavelengths) correspond to fine-scale details such as sharp cloud edges and convective cores, while low frequencies (large wavelengths) reflect broad cloud structures.

During the first three lead times (Fig. 6a–c), all models reproduce large-scale variance reasonably well, but their treatment of fine scales diverges. The 3D U-Net and ConvLSTM rapidly lose power at high frequencies, consistent with their tendency to generate overly smooth fields when trained with pixel-wise error objectives. By contrast, the 3D U-Net Diffusion model preserves substantially more small-scale variance, reflecting the ability of its generative denoising process to counteract forecast blur. The Optical Flow baseline closely matches observed spectra at fine scales in the short range, since it advects existing textures without introducing additional smoothing. However, it cannot represent dynamical evolution such as cloud growth, dissipation, or changes in morphology, limiting its fidelity at longer lead times.

At extended lead times (Fig. 6d–f), the discrepancy between forecasts and observations increases across all models, particularly at fine scales. Nonetheless, the diffusion model and Optical Flow consistently outperform 3D U-Net and ConvLSTM, which become increasingly smooth. Optical Flow remains closest to observations in spectral space at the smallest scales, underscoring the importance of texture preservation, but its inability to capture cloud evolution highlights the need for generative approaches. By combining predictive skill with improved spectral realism, the diffusion model achieves the best balance of accuracy and structural fidelity. These results provide essential complements to bulk error metrics, offering insight into the scale-dependent behavior of models and confirming that diffusion-based approaches are especially well suited for applications requiring realistic representation of fine-scale cloud features.

**Fig. 7:**
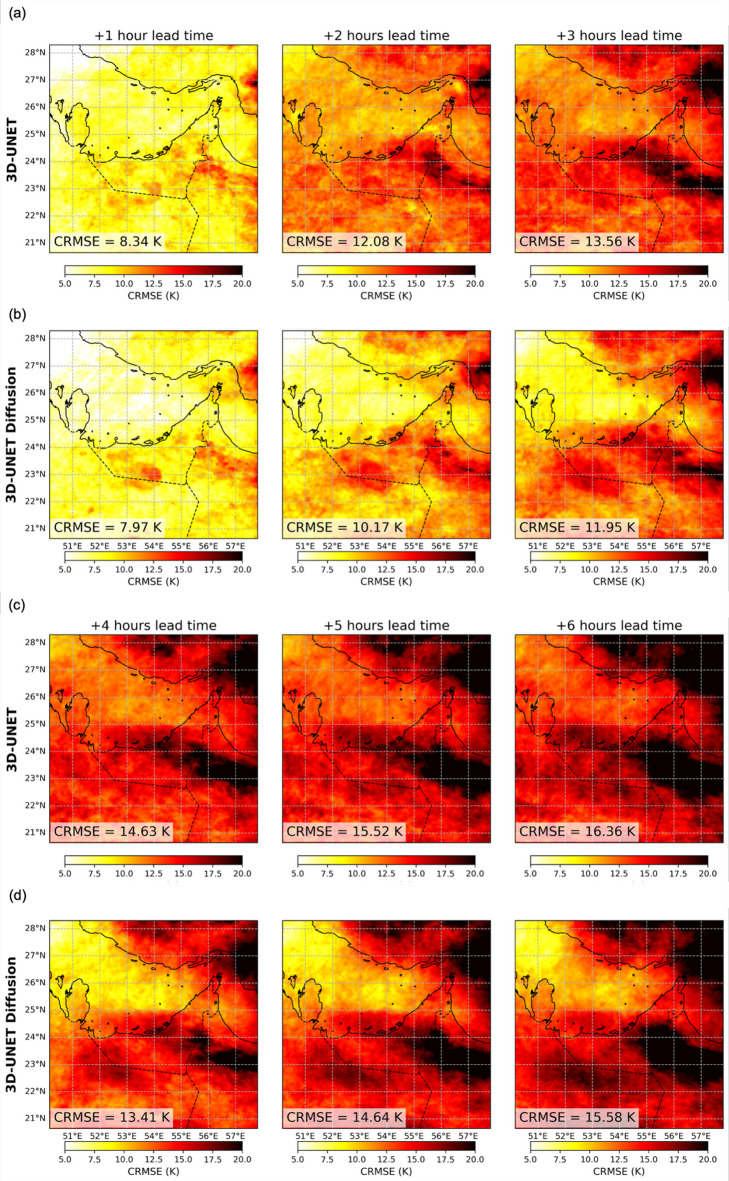
Spatial centered root-mean-square error (CRMSE) maps for the 3D U-Net and 3D U-Net Diffusion models at 1–6 h lead times. Panels (a) and (b) show CRMSE fields at 1-, 2-, and 3-hour lead times for the 3D U-Net and 3D U-Net Diffusion models, respectively. Panels (c) and (d) show the corresponding CRMSE fields at 4-, 5-, and 6-hour lead times. Each pixel represents the CRMSE over all forecasts in the July–September 2022 independent test period. The domain-averaged CRMSE for each lead time is reported in the lower-left corner of each subplot.

To further examine the spatial distribution of nowcast errors over the study domain, Fig. 7 compares spatial CRMSE for the 3D U-Net and 3D U-Net Diffusion forecasts at 1–6 h lead times. Since the performance of the 3D U-Net and 3D U-Net Diffusion models is closer than that of the other benchmarks, we show the spatial map comparisons on these two models. This allows a direct evaluation of how the diffusion framework improves the nowcast accuracy upon the baseline 3D U-Net. As shown in Fig. 7a–b, at the 1-hour lead time both models exhibit relatively low CRMSE values across the domain, though errors are concentrated over convective regions along the southeastern Arabian Peninsula. By 2–3 h, the 3D U-Net forecasts show widespread increases in CRMSE, with values exceeding 12–13 K in several convective bands, while the diffusion model maintains lower errors (~ 10–12 K) and a more spatially homogeneous error field. At longer lead times (Fig. 7c–d) CRMSE values grow substantially for both models, but the diffusion model continues to limit peak errors relative to the baseline 3D U-Net, particularly over the eastern parts of UAE and southern coastal Iran. The associated spatial distributions of bias and correlation exhibit patterns broadly consistent with the CRMSE fields and are provided in Supplementary Figures S1 and S2 for reference. These results highlight the 3D U-Net Diffusion model’s ability to preserve finer-scale Tb field structures and mitigate error growth in dynamically active regions.

**Fig. 8:**
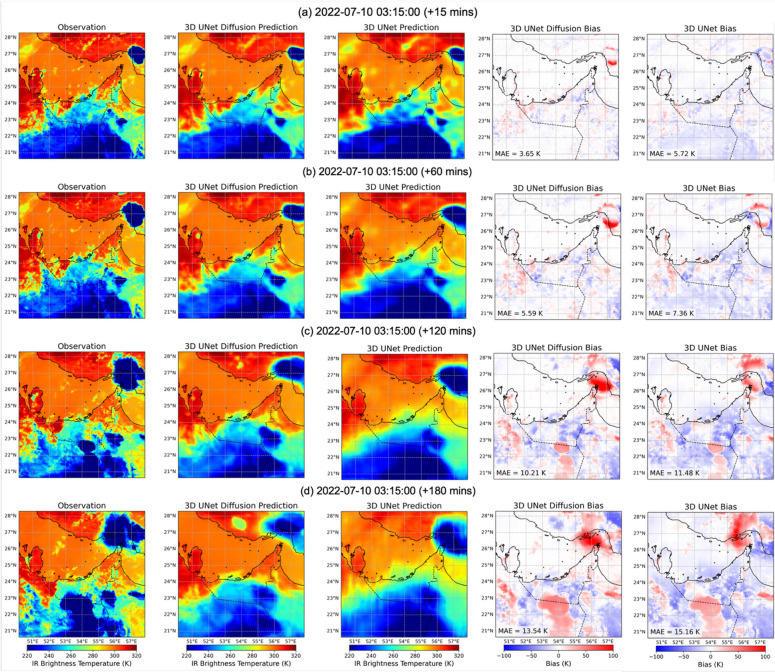
Visualization of Tbs nowcasting, using 3D U-Net Diffusion and 3D U-Net models initialized on 2022-07-10 03:15 UTC. Each row (from left to right) shows the observation, 3D U-Net Diffusion prediction, 3D U-Net prediction, 3D U-Net Diffusion biases, and 3D U-Net biases at (a) 15 min, (b) 60 min, (c) 120 min, and (d) 180 min lead times.

**Fig. 9:**
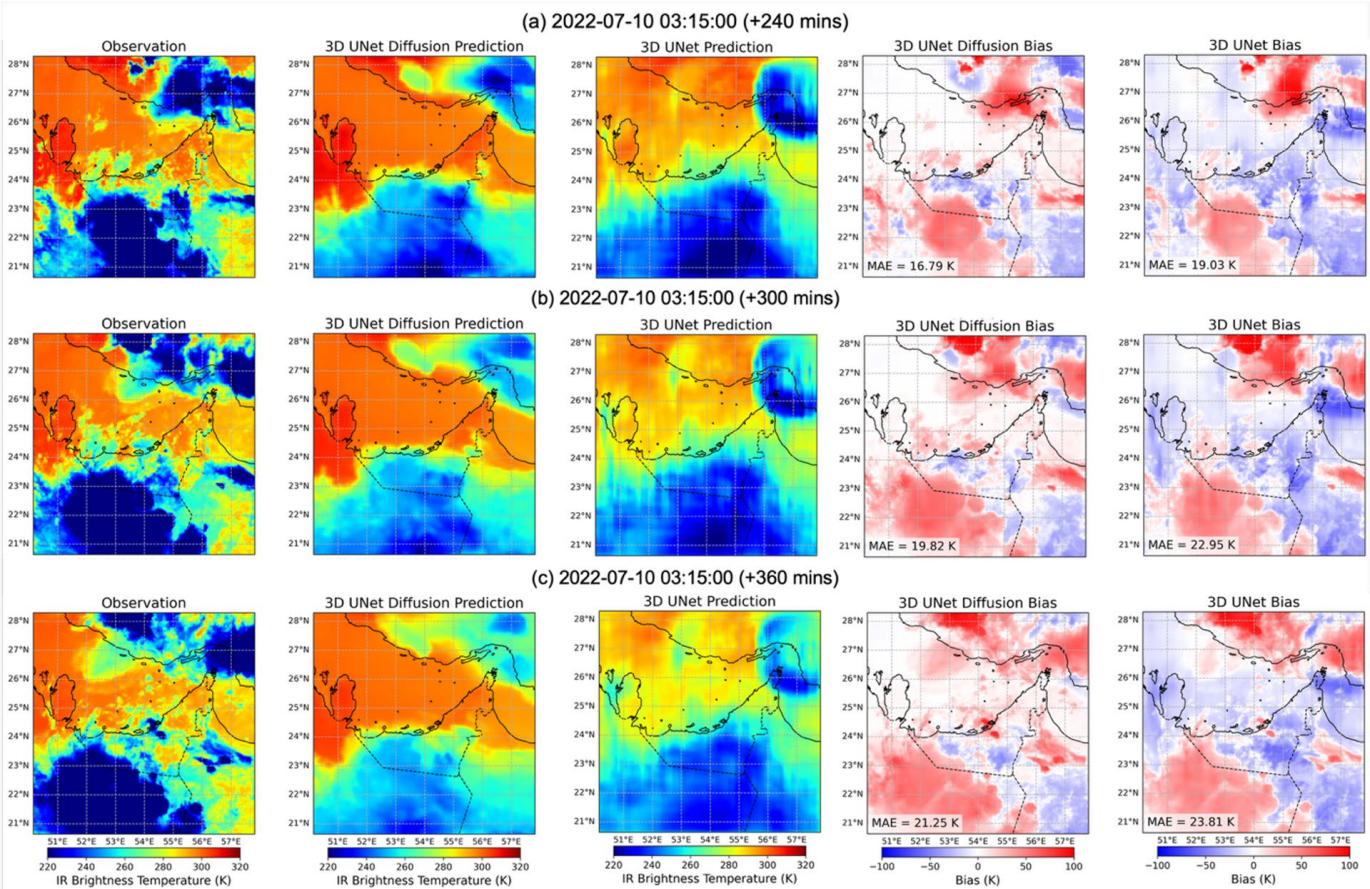
As in Fig. 8, but at (a) 240 min, (b) 300 min, and (c) 360 min lead times.

Having examined the performance of the models with different statistical metrics across the full test period, we finally present visualizations of Tb nowcasts for a representative case to illustrate how the models generate spatial Tb fields. Figures 8 and 9 present predictions initialized on 2022-07-10 03:15 UTC for lead times ranging from 15 min to 6 h. For the diffusion model, the deterministic predictions are taken as the average of nine ensembles, (see individual ensemble member as well as the member that most closely matches the observation in Supplementary Fig. S3–S9). In the short lead time range (Fig. 8a–b and 15 min), both models capture the large-scale cold Tb features, but differences in bias are already evident. The diffusion model remains nearly unbiased across most of the domain, with slight overestimation (red shading) in the northwest where a convective system is developing, with the spatial average of MAE is 3.6 K at 15 min and 5.6 K at 60 min. In contrast, the 3D U-Net shows stronger cold biases (underestimation of Tb across much of the region), with higher MAE values of 5.7 K and 7.3 K at the same lead times. By 2–3 h (Fig. 8c–d), the diffusion model exhibits growing biases linked to the emergence of deep convection, particularly over the northeastern part of the region, along with large cold Tb values in the southern sector. Interestingly, at the 3-hour lead time, the diffusion forecast produces a cold Tb patch in the central north that approximately coincides with the location of an upcoming convective system visible in the 4-hour observation (Fig. 9a), suggesting some capacity for anticipating new convection.

At longer horizons (Figs. 4, 5 and 9a–c hours), both models experience error growth, but with distinct characteristics. The diffusion model increasingly overestimates Tb, with MAE values rising from 16.8 K at 4 h to 21.3 K at 6 h. Nevertheless, its forecasts remain structurally coherent, with sharper cloud edges and more realistic placement of cold Tb features. In contrast, the 3D U-Net forecasts become progressively blurrier, with strong smoothing of cloud fields, underestimation of warm Tb regions, and overestimation of the coldest cloud tops. Its MAE values are consistently higher, growing from 19.0 K at 4 h to 23.8 K at 6 h. These differences highlight the U-Net’s tendency toward patchiness and oversmoothing at long lead times, while the diffusion model maintains better structural realism despite larger biases. It should be noted that, at lead times of 4–6 h, the deterministic 3D U-Net occasionally shows striping and checkerboard aliasing, particularly along sharp Tb gradients. We hypothesize that these artifacts arise from cumulative aliasing in the convolution upsampling stack, small grid-aligned inconsistencies introduced at each decoder stage persist and reinforce across the 24 forecast frames. By contrast, the 3D U-Net Diffusion forecasts do not show significant striping. Each frame is obtained via multiple stochastic denoising steps conditioned on the same history, so the learned spatiotemporal prior can act as a regularizer that suppresses coherent grid-aligned artifacts rather than amplifying them over time. Taken together, these case visualizations reinforce the quantitative findings: the diffusion model not only achieves lower MAE and bias on average but also produces more realistic Tb fields at the pixel level, preserving the sharpness and morphology of observed cloud systems while minimizing systematic bias across the 6-hour forecast window.

**Fig. 10:**
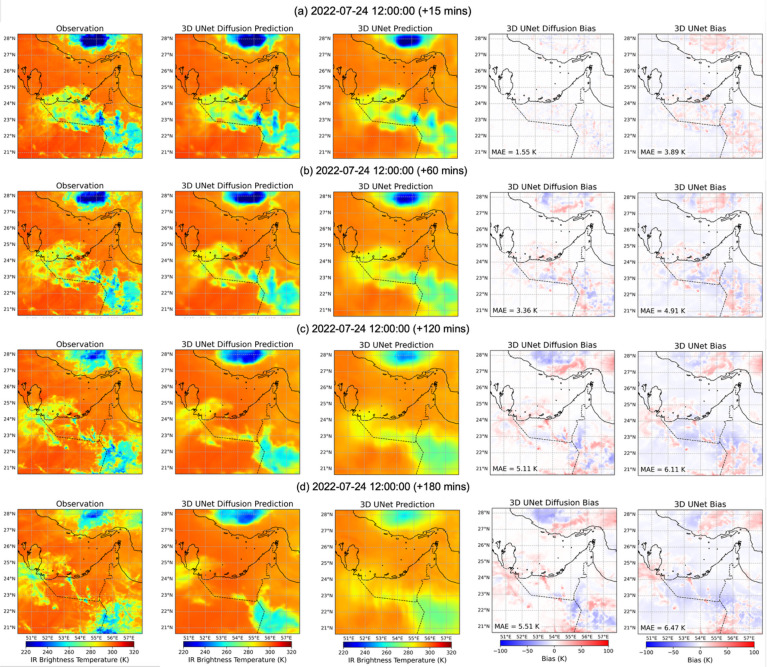
Visualization of Tbs nowcasting, using 3D U-Net Diffusion and 3D U-Net models, initialized on 2022-07-24 12:00 UTC. Each row (from left to right) shows the observation, 3D U-Net Diffusion prediction, 3D U-Net prediction, 3D U-Net Diffusion biases, and 3D U-Net biases at (a) 15 min, (b) 60 min, (c) 120 min, and (d) 180 min lead times.

**Fig. 11:**
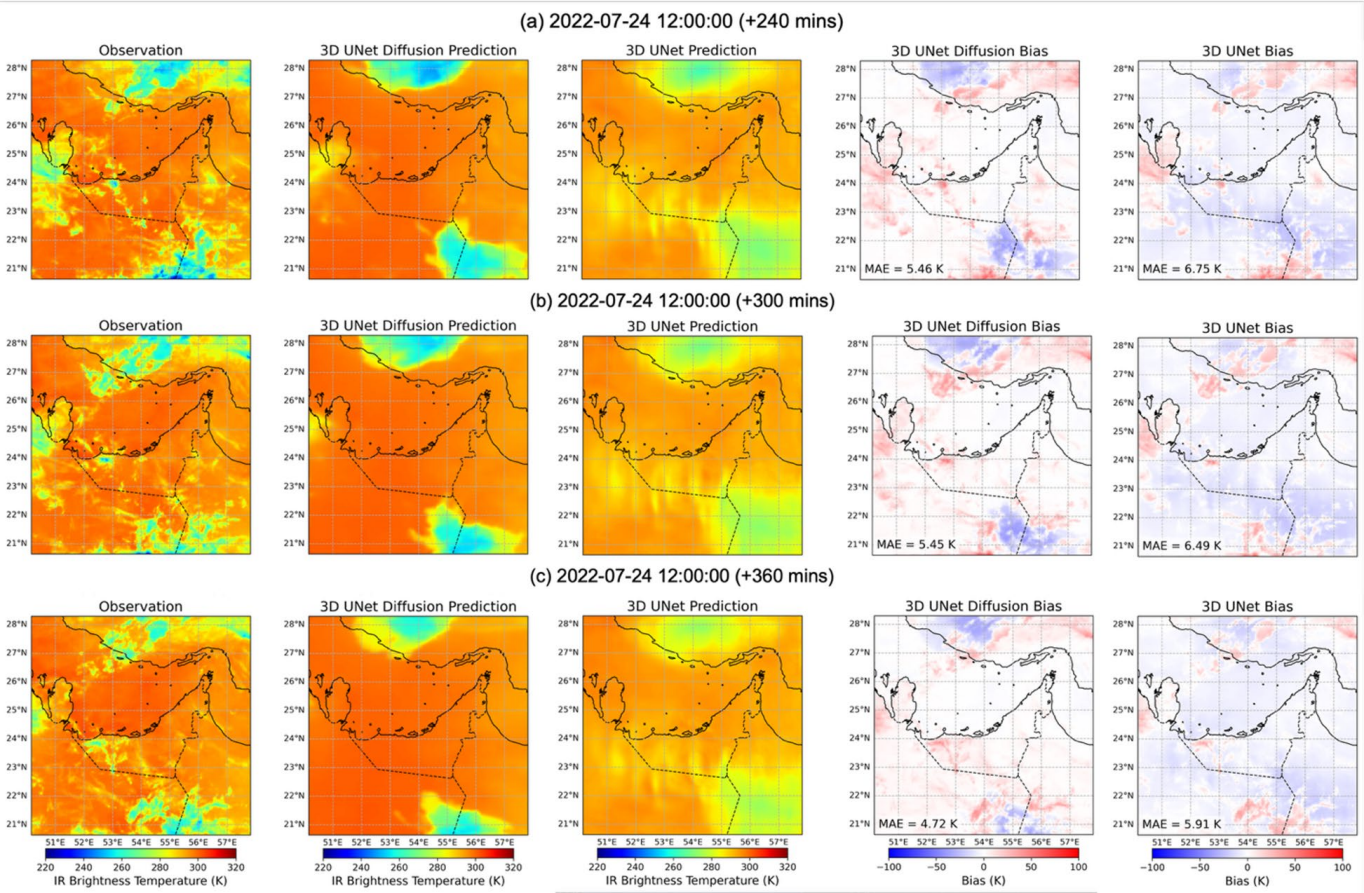
As in Fig. 10, at for (a) 240 min, (b) 300 min, and (c) 360 min lead times.

As a complementary example, we examine a second case initialized on 2022-07-24 12:00 UTC, which is characterized by generally warmer Tb values compared to the 2022-07-10 case. This case provides an opportunity to evaluate model performance when colder convective systems are less dominant and warm cloud or clear-sky conditions are more widespread across the region. Figures 10 and 11 present forecasts for lead times from 15 min to 6 h, showing observations, diffusion (see individual ensemble member in Supplementary Fig. S10–S16) and 3D U-Net predictions, and their corresponding bias fields. At short lead times (Figs. 10a–b and 15 min), both models reproduce the warm background Tb field reasonably well, with the diffusion model again showing smaller overall errors. For example, the diffusion model maintains MAE values of 1.6 K at 15 min and 3.4 K at 60 min lead time, compared to 3.9 K and 4.9 K for the U-Net, respectively. A notable feature in this period is a localized cold cloud system over the northeastern Arabian Peninsula, which the diffusion model captures with underestimation, while the U-Net overestimates its extent and magnitude, producing warm biases. By 2–3 h (Fig. 10c–d), both models begin to diverge further from the observations. The diffusion model maintains relatively modest errors (MAE = 5.1 K at 2-hour and 5.5 K at 3-hour lead time), though it slightly underpredicts warm Tb values along the southern Persian Gulf and adjacent UAE coast. The 3D U-Net shows larger biases, with MAE values exceeding 6 K, reflecting both an underestimation of warm Tb regions across the southern and western domain and oversmoothing of the smaller-scale cold patch in the northeast.

At longer lead times (Fig. 11a–c and 240–360 min), the differences between the two models persist. The diffusion model sustains MAE values of 5.5 K at 240 min, 5.5 K at 300 min, and 4.7 K at 360 min, with cold biases appearing intermittently in the northern and southern regions. In contrast, the 3D U-Net shows higher errors, with MAE values of 6.8 K, 6.5 K, and 5.9 K for the same lead times. 3D U-Net forecasts are notably smoother, failing to resolve the Tb gradients associated with warm–cold contrasts, and often underestimating the warm Tb areas. Overall, this warmer case illustrates that the diffusion model’s advantage is not limited to strongly convective environments. Even when large-scale Tb fields are dominated by warm values, the diffusion model provides more accurate and spatially coherent forecasts, capturing both the location and intensity of localized cold systems while better representing the gradients of warm Tb fields across the region.

## Conclusions and future work

We introduced a 3D U-Net diffusion framework for satellite IR Tb nowcasting and showed consistent advantages over deep-learning (3D U-Net, ConvLSTM) and traditional (optical-flow) baselines across an independent test period (July–September 2022). The3D U‑Net Diffusion ensemble mean consistently outperforms the benchmark from 15 min to over 5 h lead time, achieving lower CRMSE and higher correlation from 15 min to 2 h, with reduced but persistent gains thereafter. Beyond CRMSE and correlation metrics, diffusion model yields the highest CSI and SSIM at all leads, the lowest CRPS among all models, and RAPSD spectra that better retain high-frequency variance while capturing realistic large-scale evolution. The observed improvements in nowcast accuracy, spatial sharpness, and structural coherence validate the effectiveness of the diffusion model in overcoming traditional limitations of convolutional forecasting methods such as blurriness and temporal degradation. Notably, the enhanced performance of this approach at short lead times (up to four hours) has significant implications for operational forecasting, especially in data-sparse regions like the UAE, where satellite-derived nowcasts are essential.

Despite these promising outcomes, several areas require further investigation to optimize and operationalize the proposed model. A key limitation of the current study is its relatively short evaluation period; future work should extend testing over longer time spans, ideally overlapping with regional observational campaigns, to more robustly assess model performance under diverse meteorological conditions. Subsequent studies could also focus on extending the deterministic diffusion approach into an ensemble nowcasting framework, providing probabilistic estimates essential for decision-making. Moreover, our reliance on a single longwave IR channel constrains vertical sensitivity and limits discrimination among cloud layers. Future work should therefore incorporate additional spectral channels, such as water-vapor band, as well as other environmental variables to improve the spatiotemporal context. By integrating multi-channel inputs, the model can exploit complementary absorption features across the IR spectrum to better represent vertical cloud structure, enhance forecast realism, and improve estimates of Tb and cloud-feature characteristics.

Real-time application and verification of the developed model within operational systems presents another vital avenue for future work. This would involve integrating nowcasts into existing decision-support platforms to monitor convection, guide aviation hazard assessments, and optimize cloud-seeding operations. Systematic evaluation in a near-real-time setting would enable the assessment of real-world benefits and limitations, providing practical insights into model performance under varying meteorological scenarios. Furthermore, while the current model is a standalone satellite observation-driven approach, integrating radar data assimilation (DA) could bring it closer to operational applicability. Future work should explore hybrid AI–DA architectures, where diffusion-driven nowcasts are nudged towards DA-corrected states or vice versa, to bridge the gap towards real-time operational forecasting. Finally, extending model training to encompass a wider range of meteorological conditions, and exploring methods such as transfer learning, could further enhance model robustness and support broader applicability across regions and environmental regimes beyond the current study domain.

## Supplementary Information

Below is the link to the electronic supplementary material.

Supplementary Material 1

## Data Availability

The High Rate SEVIRI Level 1.5 Image Data are publicly available through EUMETSAT Data Services, https://navigator.eumetsat.int/start. https://navigator.eumetsat.int/product/EO: EUM: DAT: MSG: HRSEVIRI-IODC? query=High%20Rate%20SEVIRI%20Level%201.5%20Image%20Data%20-%20MSG%20-%20Indian%20Ocean&s=advanced.

## Code availability

The code implementing the video diffusion models used in this study—based on Jonathan Ho’s extension of DDPMs to video generation—was developed in PyTorch using the open-source repository at https://github.com/lucidrains/video-diffusion-pytorch. All scripts and training configurations will be made available from the corresponding author upon reasonable request.
