# Supplementary material for: Deterministic nowcasting of geostationary satellite infrared brightness temperature using 3D U-Net diffusion model
Source: Sci Rep. 2026 Jan 3;16:4191. doi: 10.1038/s41598-025-34207-9 (PMC12859110; doi:10.1038/s41598-025-34207-9)
Supplement: Supplementary file 1 — Supplementary Material 1 [file 41598_2025_34207_MOESM1_ESM.pdf]

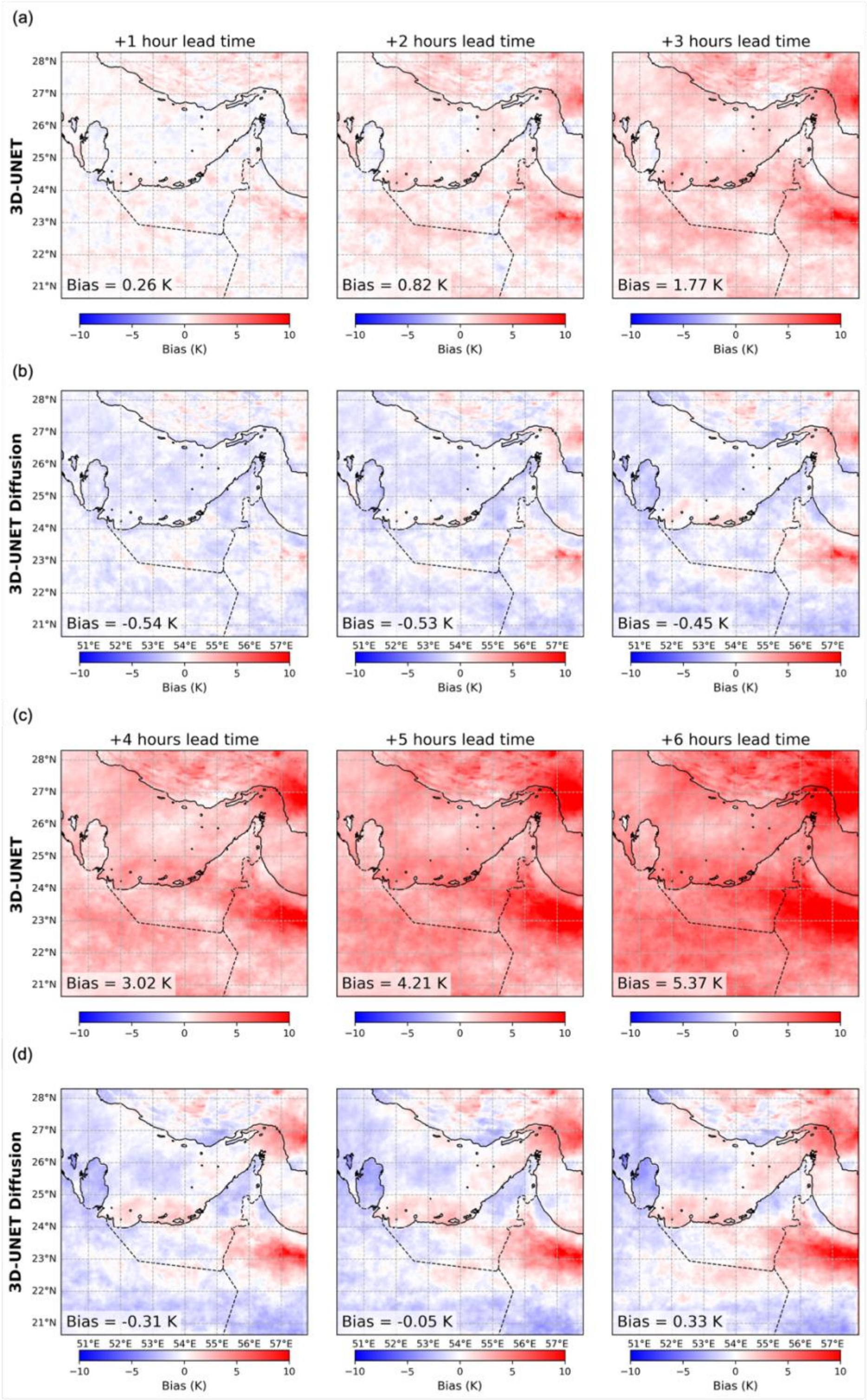

Fig S1. Spatial bias maps for the 3D U-Net and 3D U-Net Diffusion models at 1–6 h lead times. Panels (a) and (b) show CRMSE fields at 1-, 2-, and 3-hour lead times for the 3D U-Net and 3D U-Net Diffusion models, respectively. Panels (c) and (d) show the corresponding bias fields at 4-, 5-, and 6-hour lead times.

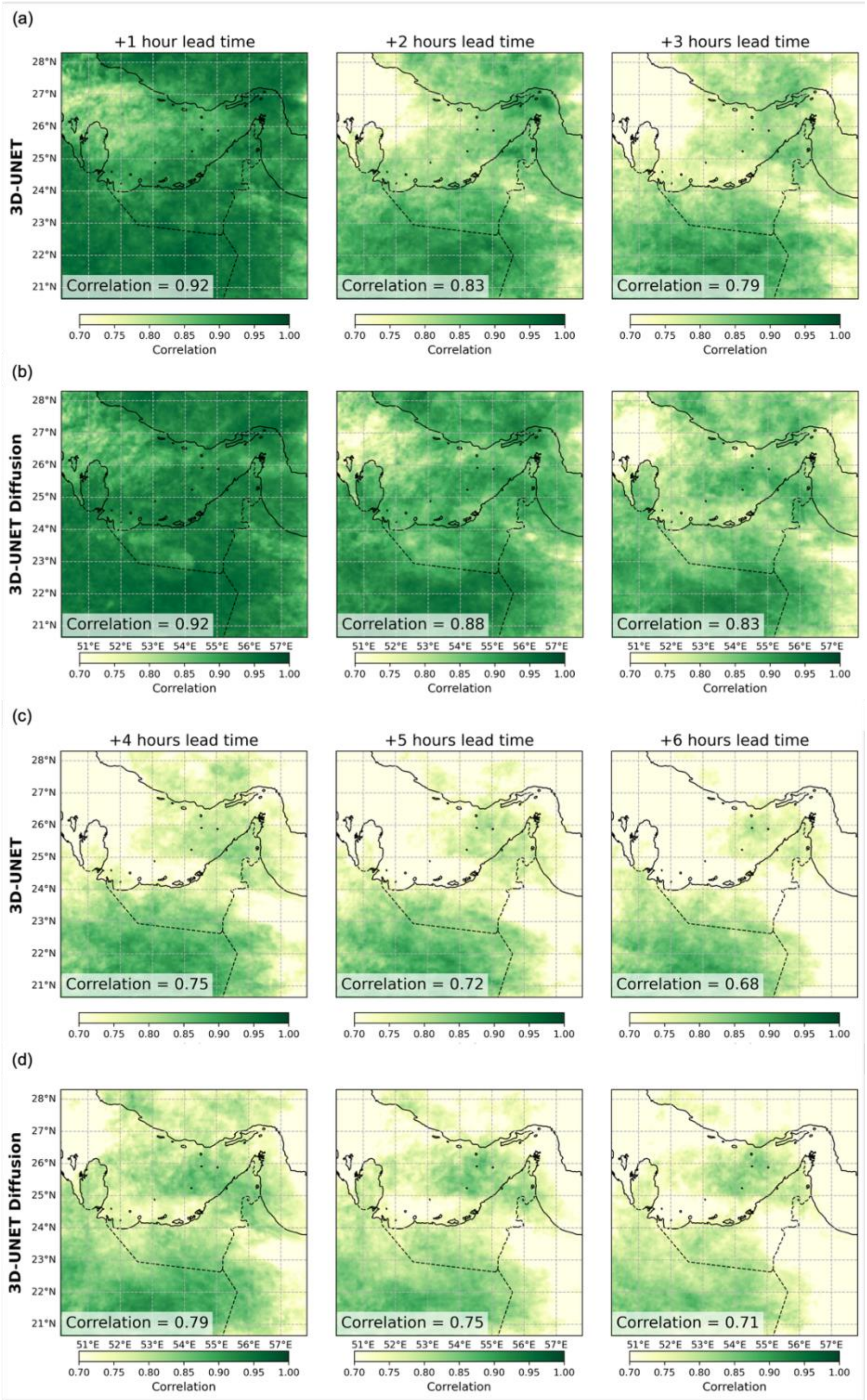

Fig S2. Spatial Pearson correlation coefficient maps for the 3D U-Net and 3D U-Net Diffusion models at 1–6 h lead times. Panels (a) and (b) show CRMSE fields at 1-, 2-, and 3-hour lead times for the 3D U-Net and 3D U-Net Diffusion models, respectively. Panels (c) and (d) show the corresponding correlation fields at 4-, 5-, and 6-hour lead times.

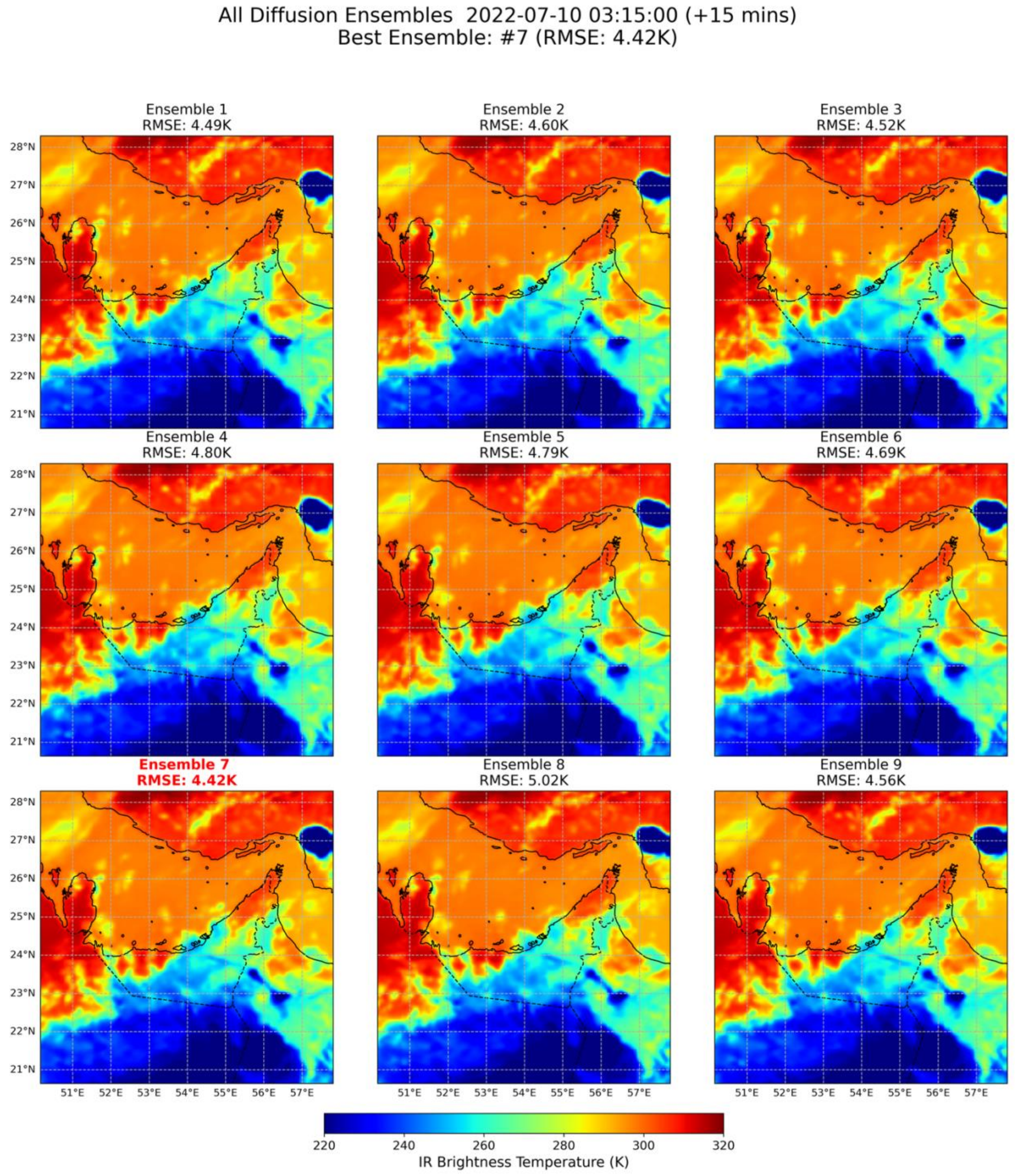

Fig S3. Diffusion ensemble members at 15 min lead time (+15 min) initialized on 2022-07-10 03:15 UTC. Nine ensemble members are shown with RMSE noted; the best-performing member is highlighted in red.

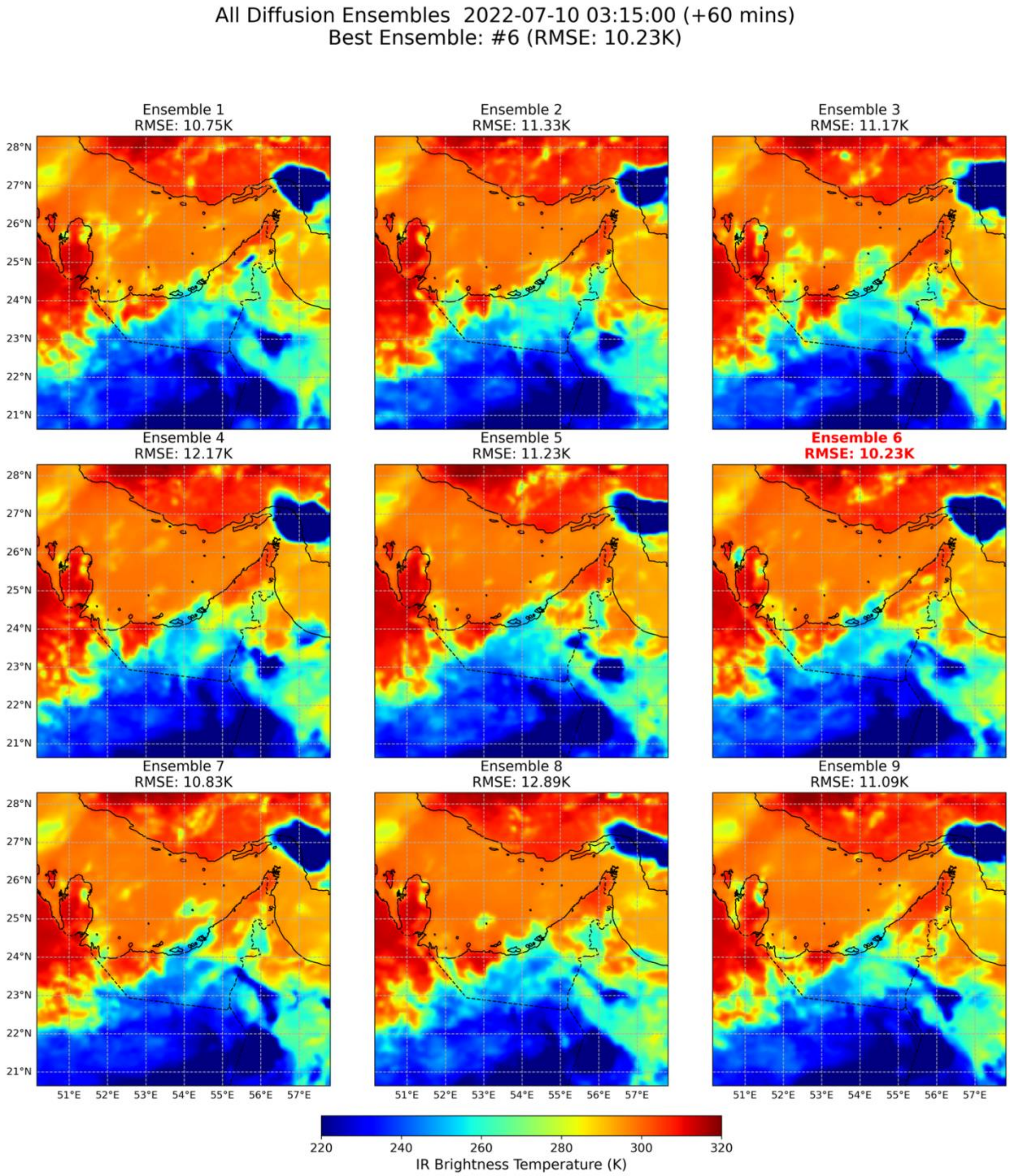

Fig S4. Diffusion ensemble members at 60 min lead initialized on 2022-07-10 03:15 UTC. Nine ensemble members are shown with RMSE noted; the best-performing member is highlighted in red.

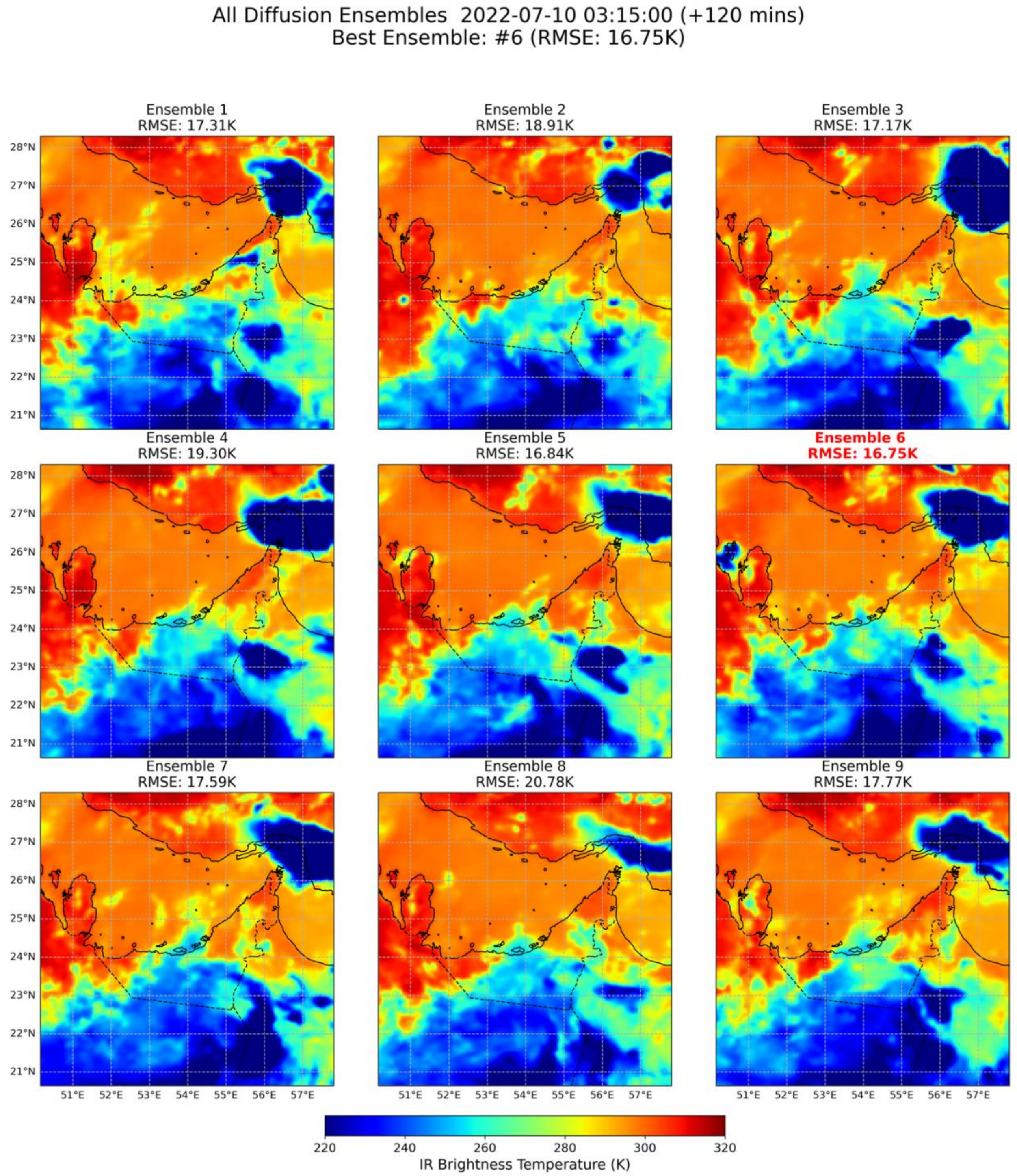

Fig S5. Diffusion ensemble members at 120 min lead time initialized on 2022-07-10 03:15 UTC. Nine ensemble members are shown with RMSE noted; the best-performing member is highlighted in red.

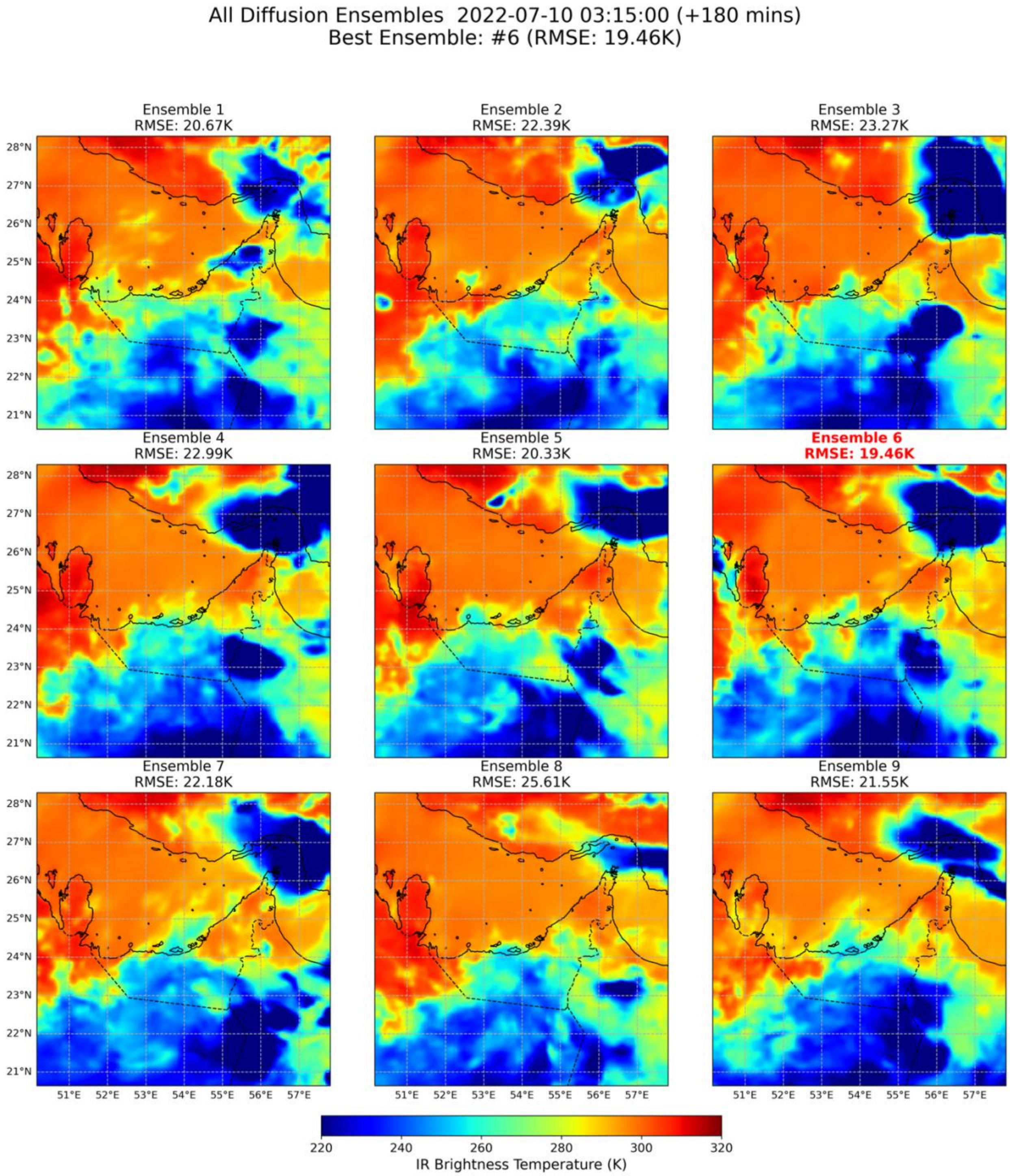

Fig S6. Diffusion ensemble members at 180 min lead time initialized on 2022-07-10 03:15 UTC. Nine ensemble members are shown with RMSE noted; the best-performing member is highlighted in red.

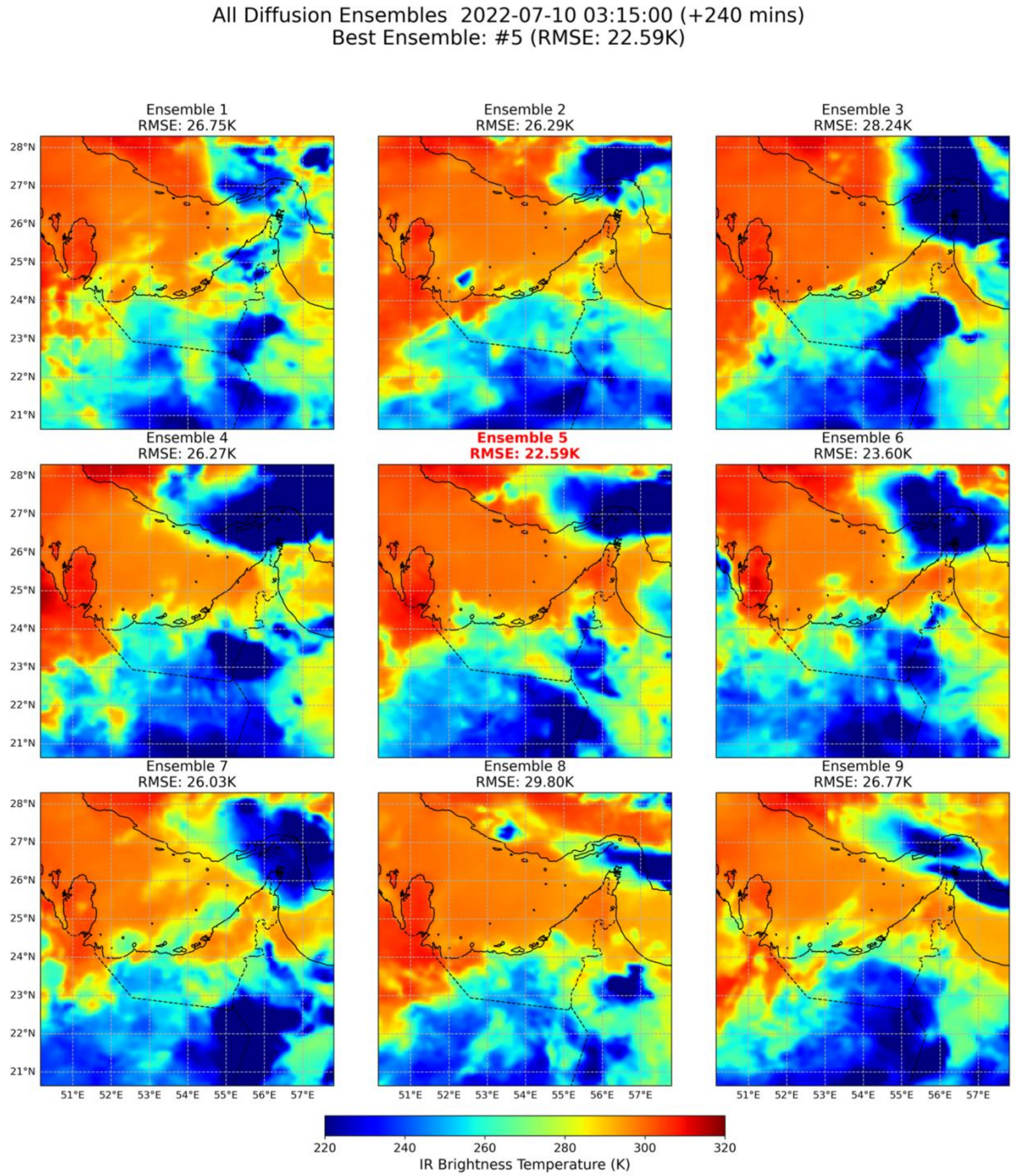

Fig S7. Diffusion ensemble members at 240 min lead time initialized on 2022-07-10 03:15 UTC. Nine ensemble members are shown with RMSE noted; the best-performing member is highlighted in red.

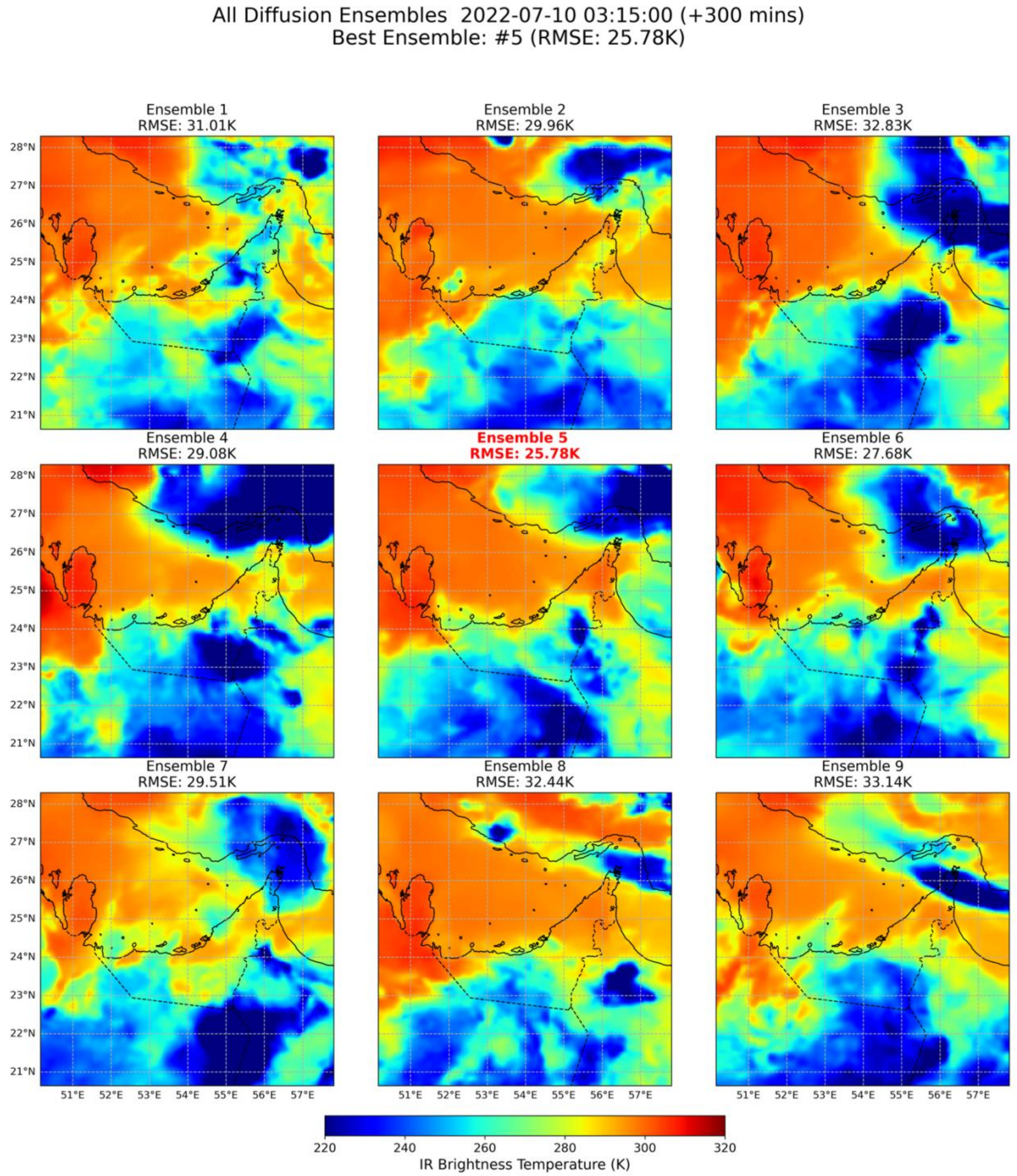

Fig S8. Diffusion ensemble members at 300 min lead time initialized on 2022-07-10 03:15 UTC. Nine ensemble members are shown with RMSE noted; the best-performing member is highlighted in red.

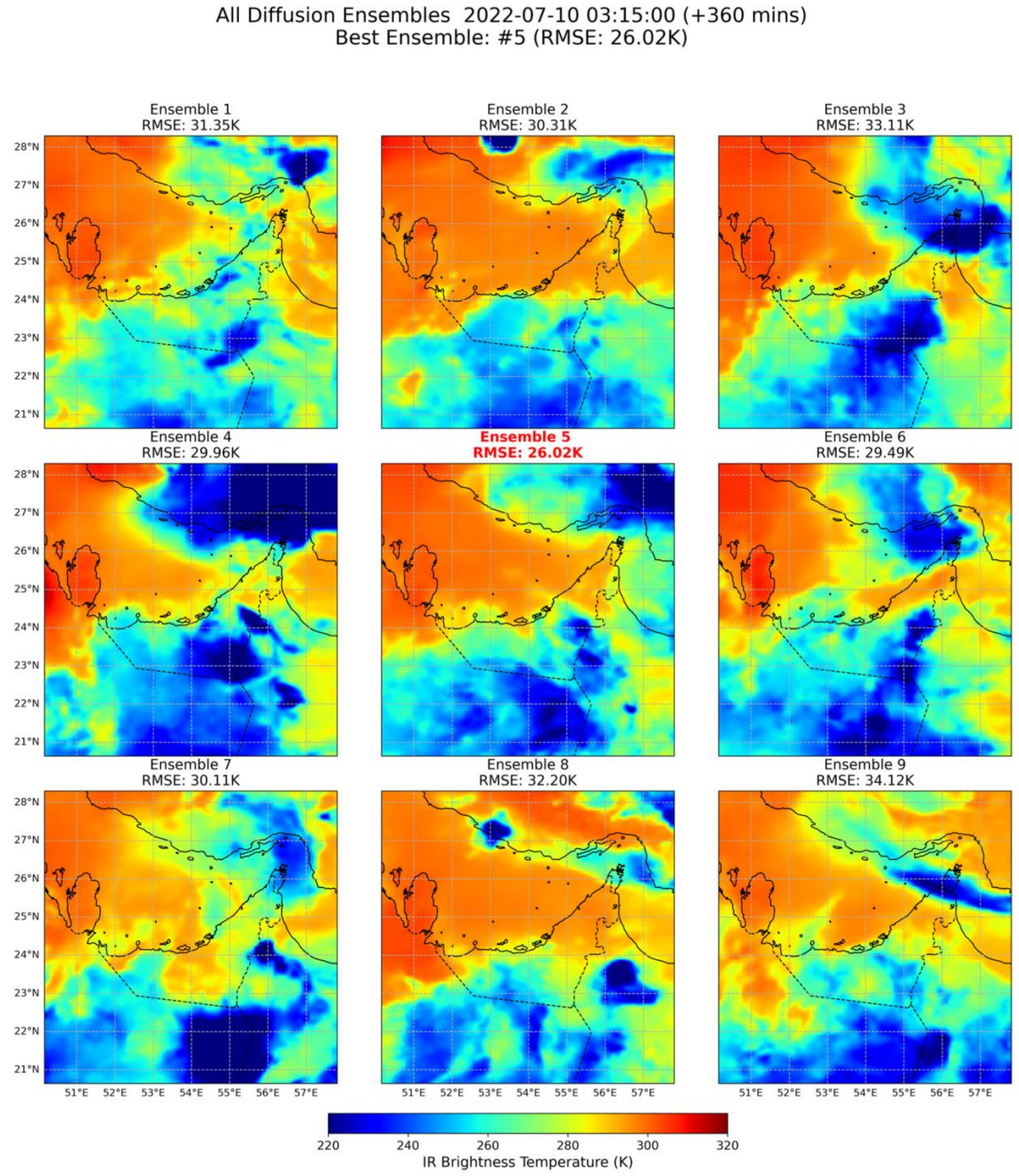

Fig S9. Diffusion ensemble members at 360 min lead time initialized on 2022-07-10 03:15 UTC. Nine ensemble members are shown with RMSE noted; the best-performing member is highlighted in red.

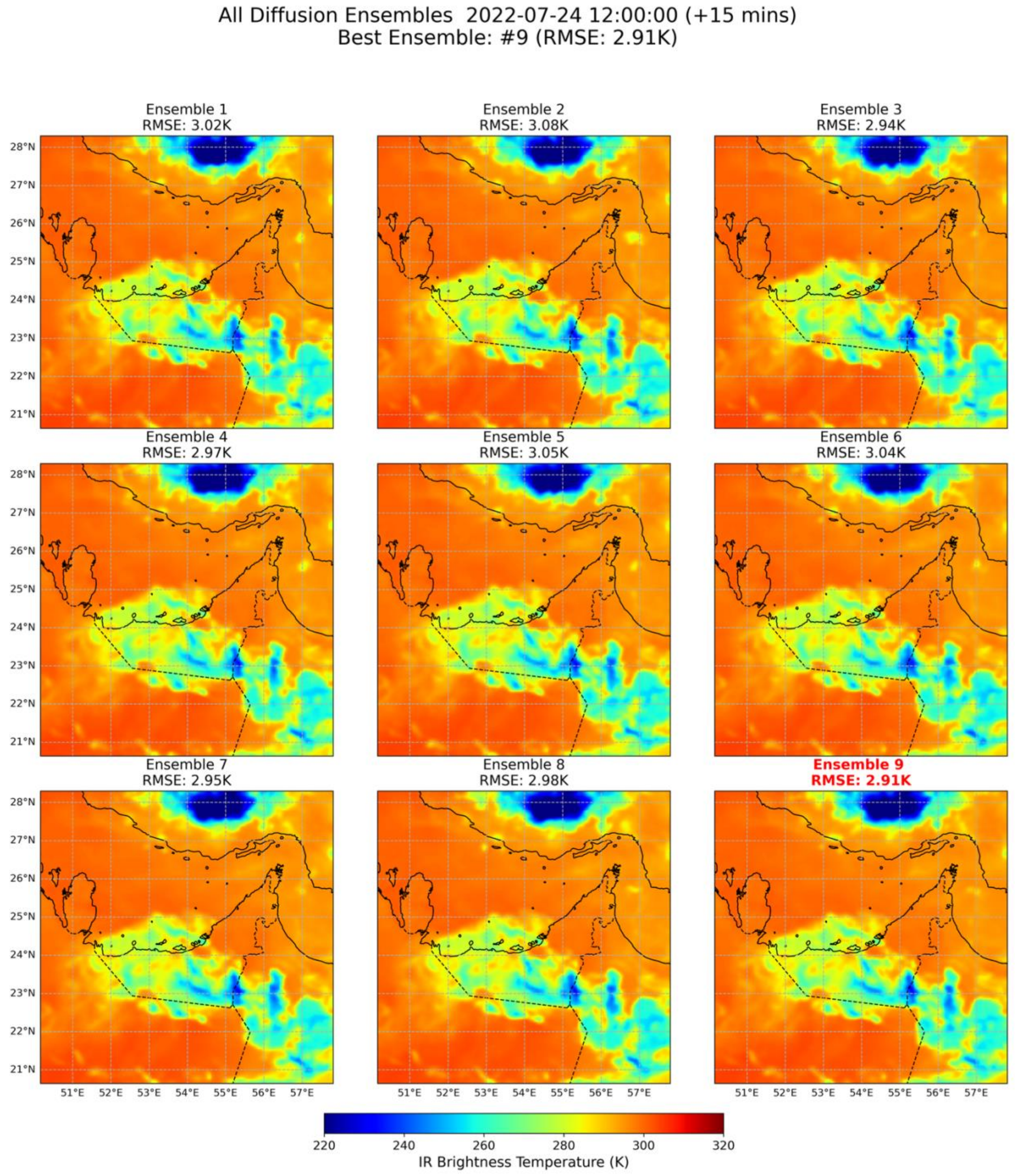

Fig S10. Diffusion ensemble members at 15 min lead time initialized on 2022-04-24 12:00 UTC. Nine ensemble members are shown with RMSE noted; the best-performing member is highlighted in red.

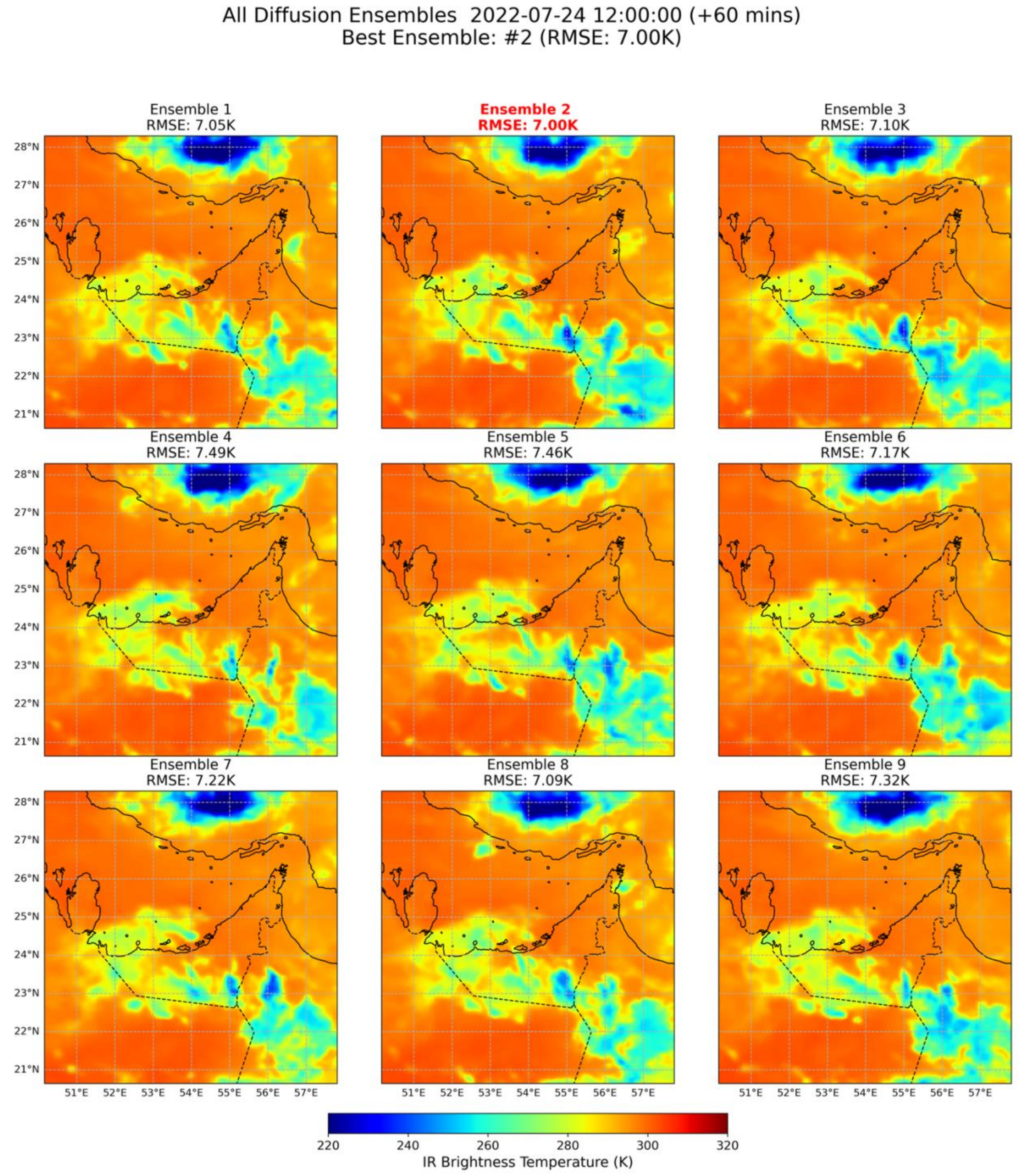

Fig S11. Diffusion ensemble members at 60 min lead time initialized on 2022-04-24 12:00 UTC. Nine ensemble members are shown with RMSE noted; the best-performing member is highlighted in red.

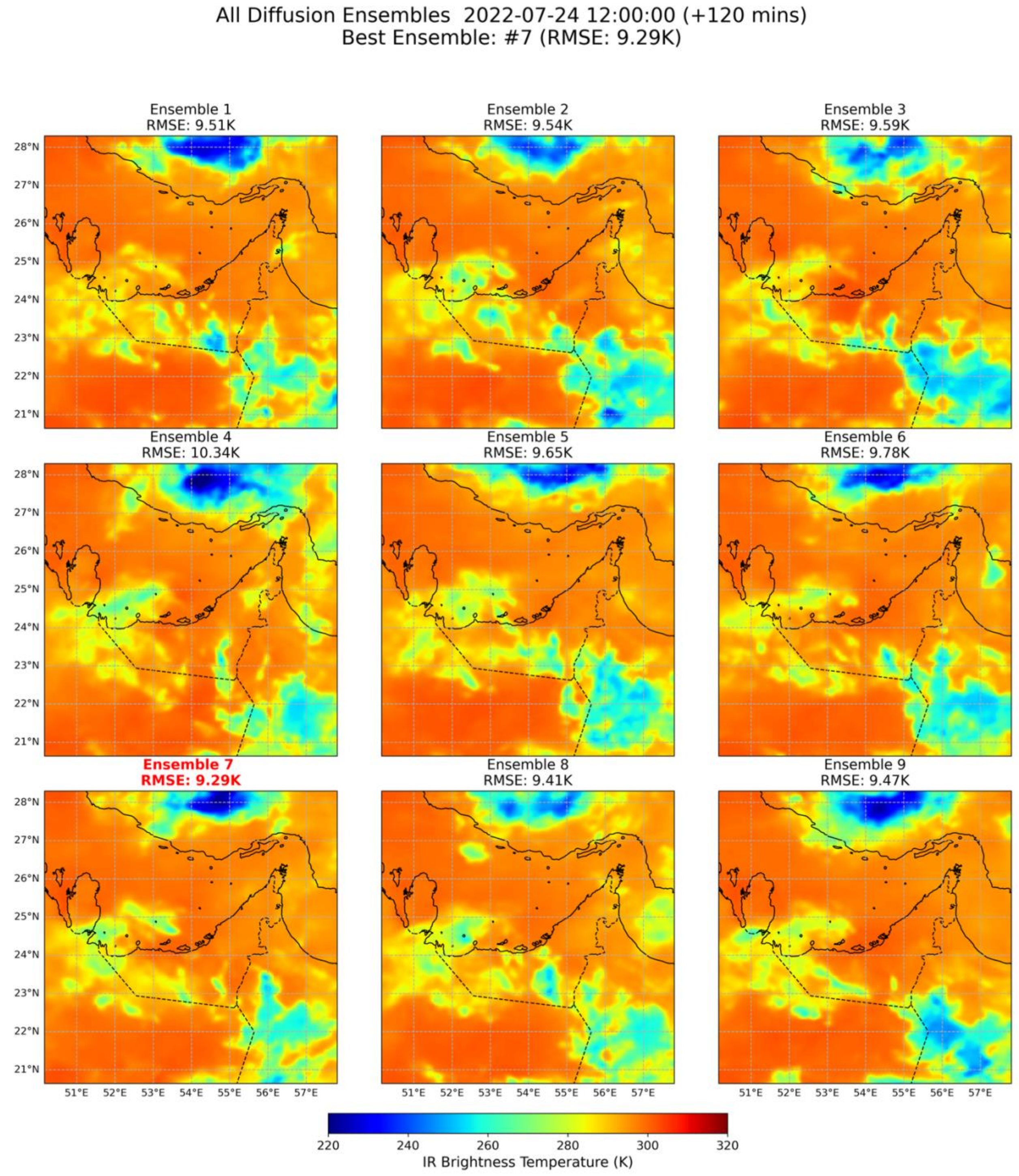

Fig S12. Diffusion ensemble members at 120 min lead time initialized on 2022-04-24 12:00 UTC. Nine ensemble members are shown with RMSE noted; the best-performing member is highlighted in red.

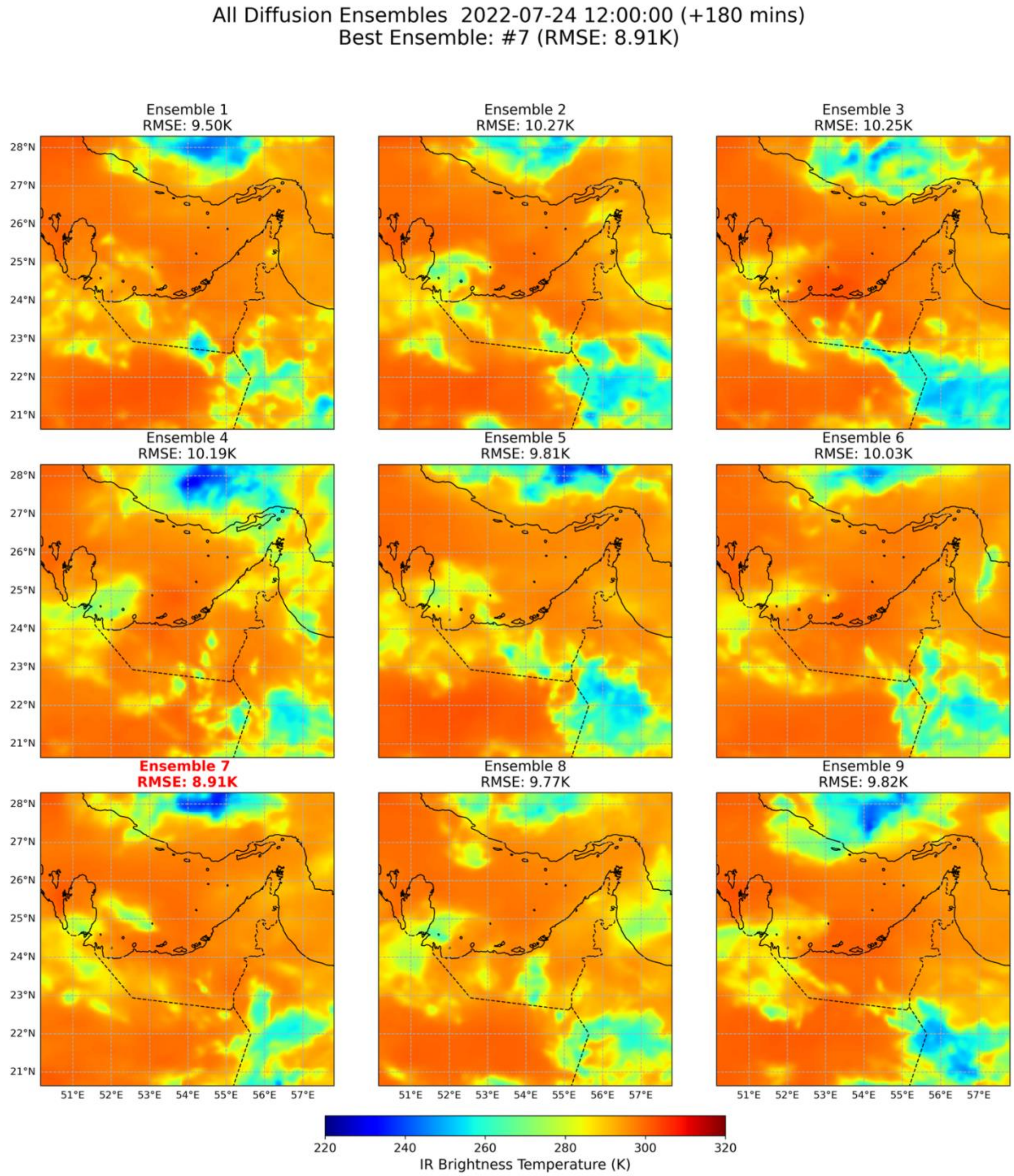

Fig S13. Diffusion ensemble members at 180 min lead time initialized on 2022-04-24 12:00 UTC. Nine ensemble members are shown with RMSE noted; the best-performing member is highlighted in red.

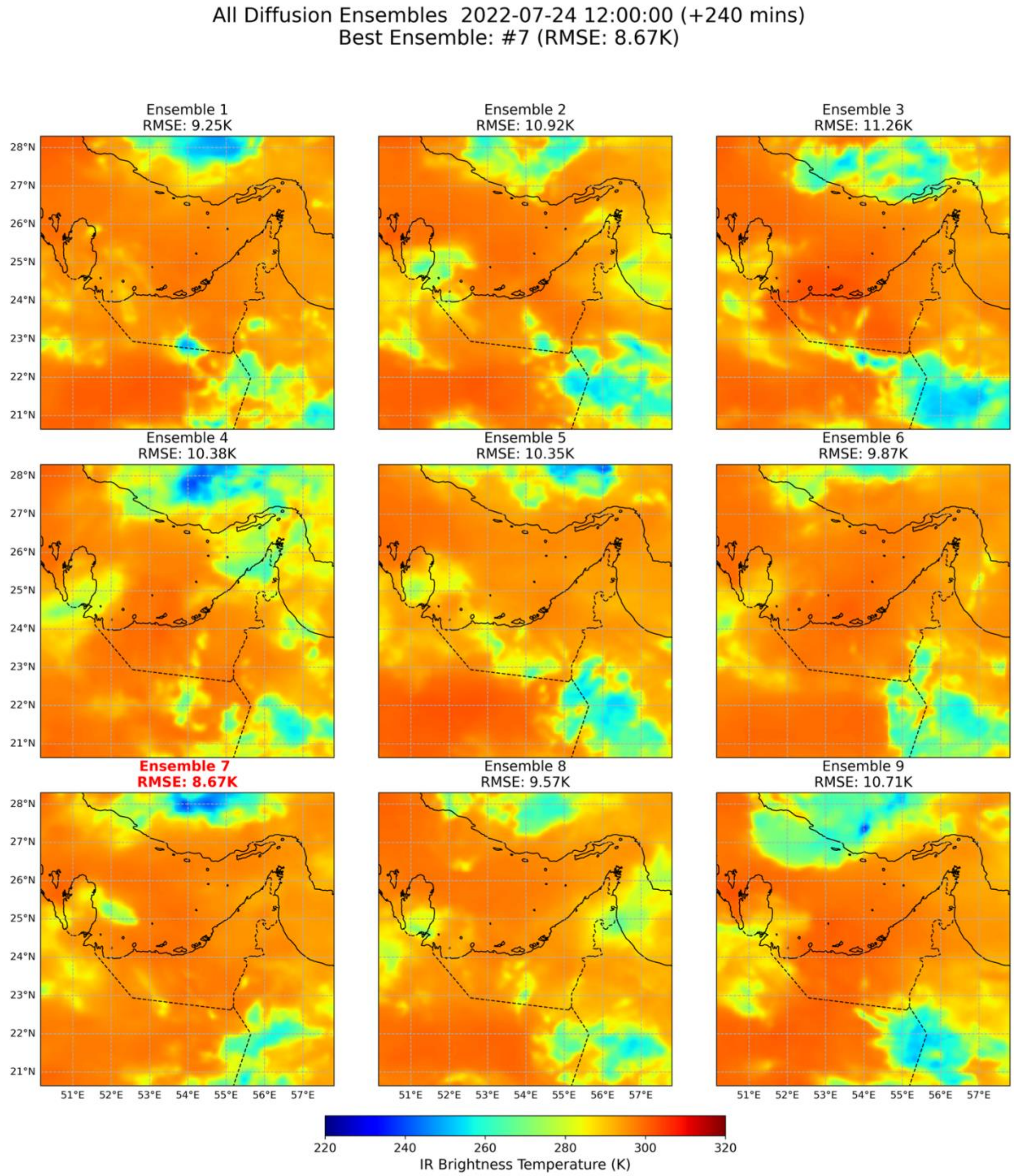

Fig S14. Diffusion ensemble members at 240 min lead time initialized on 2022-04-24 12:00 UTC. Nine ensemble members are shown with RMSE noted; the best-performing member is highlighted in red.

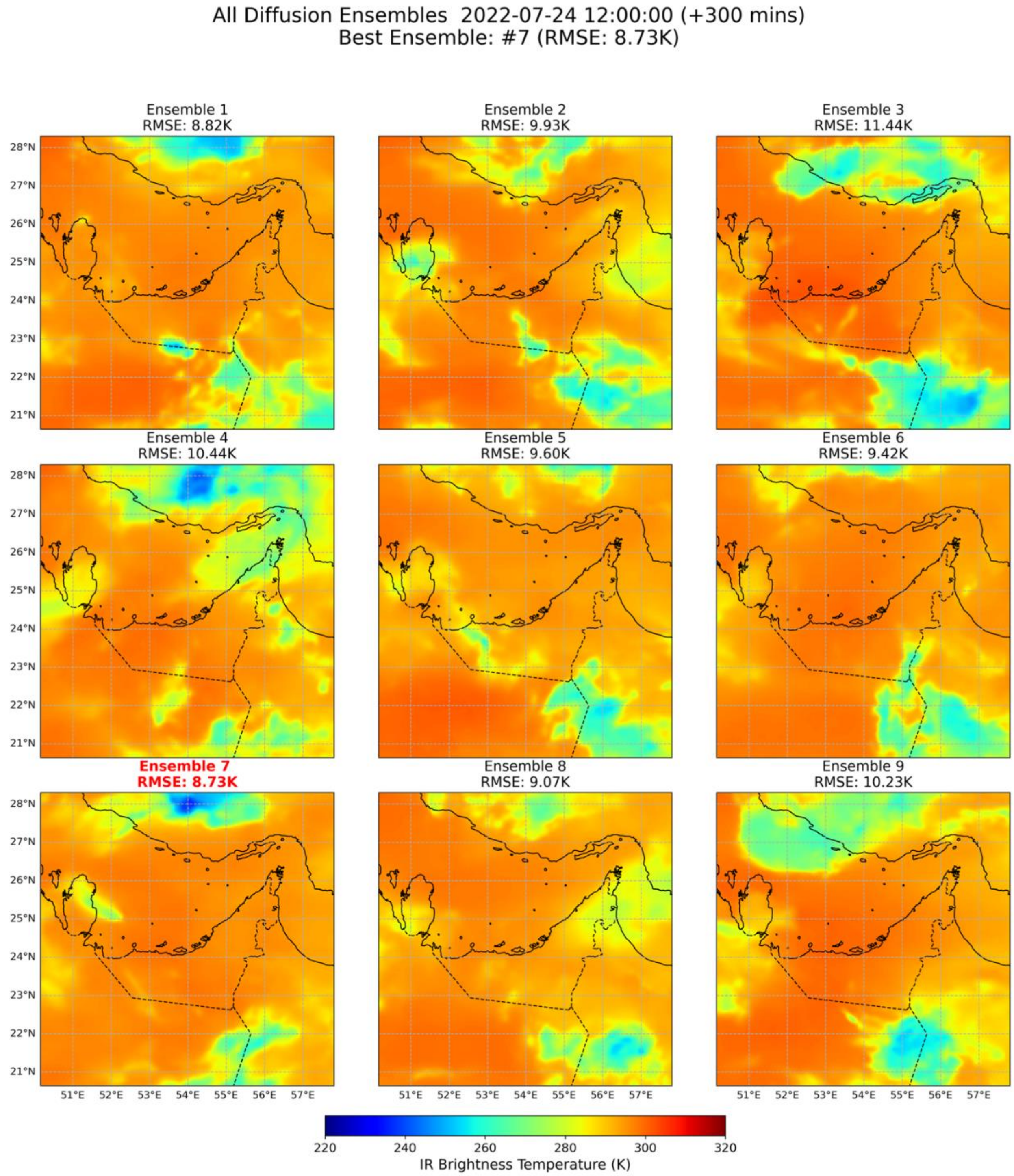

Fig S15. Diffusion ensemble members at 300 min lead time initialized on 2022-04-24 12:00 UTC. Nine ensemble members are shown with RMSE noted; the best-performing member is highlighted in red.

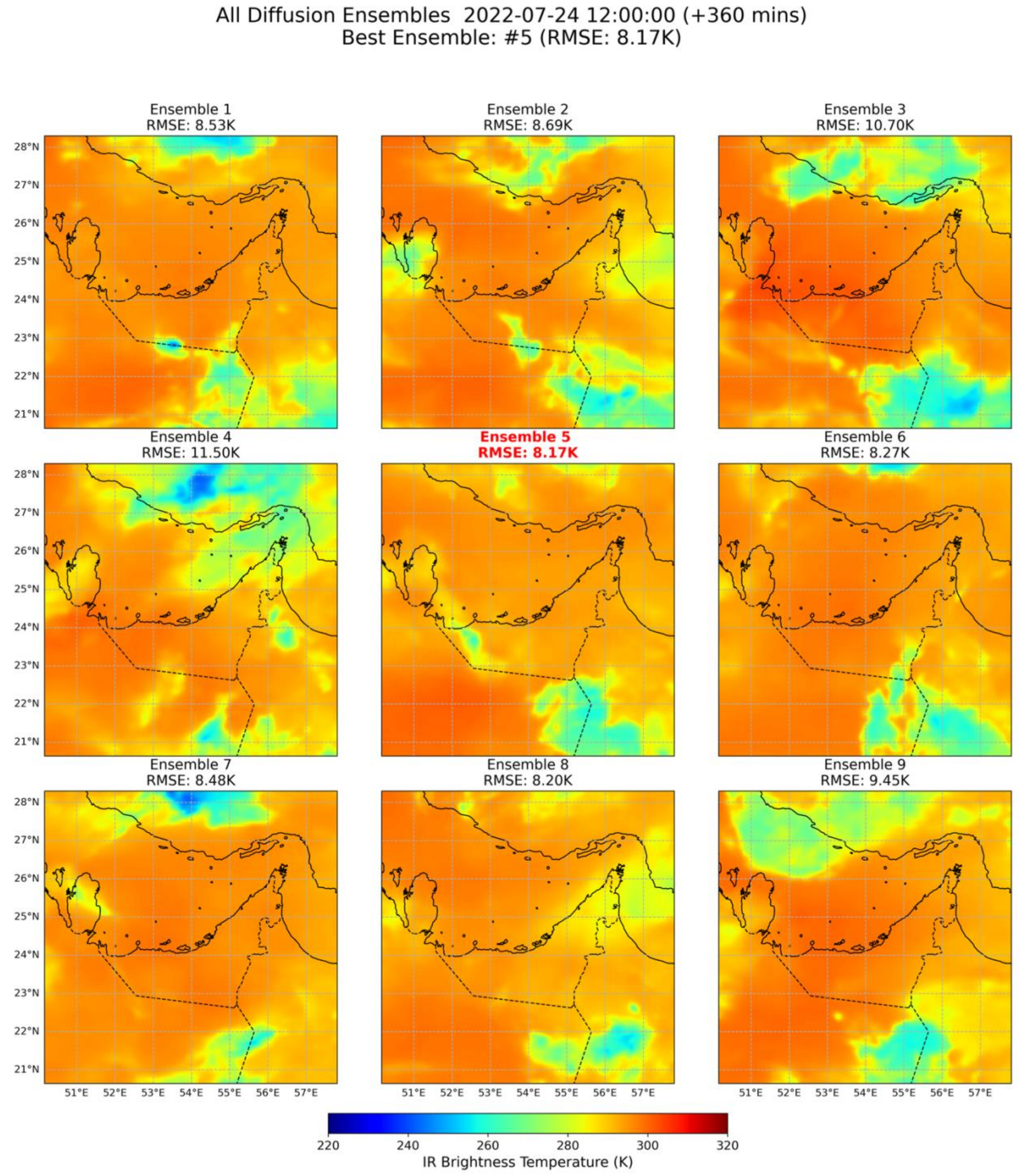

Fig S16. Diffusion ensemble members at 360 min lead time initialized on 2022-04-24 12:00 UTC. Nine ensemble members are shown with RMSE noted; the best-performing member is highlighted in red.
